# Prognostic value of TP53 concurrent mutations for EGFR- TKIs and ALK-TKIs based targeted therapy in advanced non-small cell lung cancer: a meta-analysis

**DOI:** 10.1186/s12885-020-06805-5

**Published:** 2020-04-16

**Authors:** Kang Qin, Helei Hou, Yu Liang, Xiaochun Zhang

**Affiliations:** Department of Medical Oncology, The Affiliated Hospital of Qingdao University, 16 Jiangsu Road, Qingdao, 266005 Shandong Province China

**Keywords:** Tumor protein 53, Non-small-cell lung cancer, Epidermal growth factor receptor, Anaplastic lymphoma kinase, Tyrosine kinase inhibitors

## Abstract

**Background:**

The prognostic significance of TP53 concurrent mutations in patients with epidermal growth factor receptor (EGFR)- or anaplastic lymphoma kinase (ALK)- mutated advanced non–small-cell lung cancer (NSCLC) who received EGFR-tyrosine kinase inhibitors (TKIs) or ALK-TKIs based targeted therapy remains controversial. Therefore, the present meta-analysis was performed to investigate the association between TP53 concurrent mutations and prognosis of patients with advanced NSCLC undergoing EGFR-TKIs or ALK-TKIs treatments.

**Methods:**

Eligible studies were identified by searching the online databases PubMed, Embase, Medline, The Cochrane library and Web of Science. Hazard ratios (HRs) with 95% confidence intervals (CIs) were calculated to clarify the correlation between TP53 mutation status and prognosis of patients. This meta-analysis was conducted according to the Preferred Reporting Items for Systematic Reviews and Meta-Analyses (PRISMA) statement.

**Results:**

In total, 15 studies with 1342 patients were included for final analysis. Overall, concurrent TP53 mutation was associated with unfavorable progression-free survival (PFS) (HR = 1.88, 95%CI: 1.59–2.23, *p* < 0.001, I^2^ = 0.0%, *P* = 0.792) and overall survival (OS) (HR = 1.92, 95%CI: 1.55–2.38, *p* < 0.001, I^2^ = 0.0%, *P* = 0.515). Subgroup analysis based on type of targeted therapy (EGFR-TKIs or ALK-TKIs, pathological type of cancer (adenocarcinoma only or all NSCLC subtypes) and line of treatment (first-line only or all lines) all showed that TP53 mutations was associated with shorter survivals of patients with EGFR-TKIs or ALK-TKIs treatments. Particularly, in patients with first-line EGFR-TKIs treatment, significantly poorer prognosis was observed in patients with TP53 concurrent mutations (pooled HR for PFS: 1.69, 95% CI 1.25–2.27, *P* < 0.001, I^2^ = 0.0%, *P* = 0.473; pooled HR for OS: 1.94, 95% CI 1.36–2.76, *P* < 0.001, I^2^ = 0.0%, *P* = 0.484). Begg’s funnel plots and Egger’s tests indicated no significant publication bias in this study.

**Conclusions:**

This meta-analysis indicated that concurrent TP53 mutations was a negative prognostic factor and associated with poorer outcomes of patients with EGFR-TKIs or ALK-TKIs treatments in advanced NSCLC. In addition, our study provided evidence that TP53 mutations might be involved in primary resistance to EGFR-TKIs treatments in patients with sensitive EGFR mutations in advanced NSCLC.

## Background

Non-small cell lung cancer (NSCLC) constitutes 85 to 90% of lung cancer and is the leading cause of cancer mortality worldwide [1].Clinically, over 60% of patients with NSCLC were diagnosed with advanced or metastatic disease (stage III or IV), at which point surgical resection is not a workable choice, resulting in a 5-year survival rate lower than 20% [2].In the past decade, great advances in understanding of molecular properties and the immune microenvironment have made targeted therapy and immunotherapy the optimal treatment paradigms for patients with advanced NSCLC harboring activating driver gene mutations and high level of PD-L1/PD-1 expression, which has much significantly improved prognosis and clinical outcomes of these patients compared with conventional chemotherapy and radiotherapy [3–9].

In advanced NSCLC, biomarkers recommended for assessment by the most recent version of the NCCN (National Comprehensive Cancer Network) guidelines (v02.2020).

To guide treatment selection include EGFR mutations, ALK fusions, BRAF V600E mutation, ROS1 fusions, RET fusions, MET amplification or MET exon 14 skipping variants, ERBB2 (HER2) mutations, NTRK fusions, PD-L1 expression and tumor mutational burden (TMB) level (https://www.nccn.org/professionals/physician). For those patients with advanced NSCLC and positive activating driver mutations, first-line treatment recommended is the corresponding targeted drugs. For those who have high level of PD-L1 expression (≥50%) and negative actionable driver mutations, immunotherapy with PD-1/PD-L1 checkpoint inhibitors is the treatment on first choice (on condition that there is no contraindications to PD-1 or PD-L1 checkpoint inhibitors). For patients with advanced NSCLC and present with a respectively lower level of PD-1/PD-L1 expression (ranging from 1 to 49%) and negative targetable mutations, combined treatment with platin-based chemotherapy and immunotherapy is the first-class therapeutic strategy.

Among all targetable biomarkers in advanced NSCLC as mentioned above, the epidermal growth factor receptor (EGFR) activating mutations and the anaplastic lymphoma kinase (ALK) rearrangements are two main families of targetable oncogenic mutations, which could be been found in 20–50% and 3–7% of all NSCLC patients respectively [10–13]. Targeted drugs recommended by NCCN guidelines for treatment of patients with oncogenic EGFR mutations (mainly exon 19 deletions and exon 21 point mutations) include gefitinib, erlotinib, icotinib, afatinib and osimertinib, ect [3, 4]. Crizotinib was the first ALK-TKI approved for targeted treatment of patients with ALK rearranged-NSCLC (mainly EML4-ALK fusions), followed by next generation ALK-TKIs (including ceritinib, brigatinib, alectinib, and lorlatinib) based on a dramatic improvement in clinical outcome and prognosis of patients compared with traditional chemotherapies [14, 15].

However, in EGFR or ALK-mutated advanced NSCLC, although the majority of patients demonstrated excellent and durable response to EGFR-TKIs or ALK-TKIs, almost all patients will undergo relapse and disease progression within 1-2 years after treatment initiation [16, 17]. Approximately 50% of secondary resistance to EGFR-TKIs is due to EGFR exon 20 T790M mutation [16, 17] and 37% of secondary resistance to ALK-TKIs is attributed to ALK secondary mutations (L1196M, L1152R, G1202R, G1269A, C1156Y etc) or amplifications [18–20]. In addition to acquired resistance, approximately 10–20% of patients with advanced NSCLC harboring EGFR sensitive mutations and 5% of patients with ALK rearrangements showed primary resistance to first line EGFR-TKIs or ALK-TKIs treatments and presented with early disease progression (typically within half years after the first treatment) [11, 16–20]. Mechanisms of primary resistance to EGFR-TKIs or ALK-TKIs remained largely unknown. It was hypothesized that MET amplification, BIM polymorphisms, PIK3CA mutations, RB1 mutation, AKT1 amplification; HGF amplification, and alterations of the PIK3CA/AKT/mTOR pathway were associated with primary resistance to EGFR-TKIs in patients with advanced EGFR mutated-NSCLC [21]. Meanwhile, over-activation of the ALK signaling such as ALK amplification, up-regulation of bypass signaling pathways might be chief culprits that confer primary resistance to ALK-TKIs [19, 20].In NSCLC, TP53 mutations are the most common co-occurring events with driver oncogenes. According to previous studies, TP53 mutations could be found in 30–72% of all EGFR-mutated NSCLCs and 25–56% of ALK-positive NSCLCs, predominantly in smokers [22, 23] and the younger patients [24]_,_ but were rare in patients with other less common targeted mutations like KRAS mutation/ROS1 re-arrangement/RET re-arrangement [25].

Previous studies have suggested that TP53 concurrent mutation might adversely impact the survival of patients with advanced NSCLC treated with EGFR-TKIs or ALK-TKIs for oncogenic EGFR or ALK mutations [26–29]. In addition, some preclinical studies indicated that TP53 might be one of the mechanisms that potentially confer resistance to EGFR- and ALK-TKIs treatment [30, 31]. However, several other studies found no correlation between TP53 mutations and survival of patients with EGFR-mutated advanced NSCLC and treated with EGFR-TKIs [32–34]. For example, in study by Labbe et al., among sixty patients who received first-generation EGFR-TKIs for advanced disease, neither objective response rate (ORR) nor progression-free survival (PFS) was significantly influenced by TP53 mutations [33].

Therefore, we performed this meta-analysis to investigate the prognostic and predictive values of TP53 mutations for outcome of patients with patients with EGFR- or ALK mutated advanced NSCLC and treated with EGFR-TKIs and ALK-TKIs based targeted therapy.

## Methods

### Literature search

All relevant articles were retrieved by searching the PubMed, Embase, Medline, The Cochrane library and Web of Science (up to May 15, 2019) using a combination of the terms ‘TP53’, ‘p53 protein’, ‘Tumor Suppressor p53’, ‘p53’ ‘mutation’, ‘non-small-cell lung cancer’, ‘NSCLC’, ‘lung cancer’, ‘lung adenocarcinoma’, ‘tyrosine kinase inhibitor’, ‘targeted therapy’, ‘TKIs’, ‘EGFR’, ‘ALK’. Two investigators (Kang Qin and Helei Hou) performed the searches independently of each other. This study was performed according to the Preferred Reporting Items for Systematic Reviews and Meta-Analyses (PRISMA) guidelines [35] (http://www.prisma-statement.org).

### Selection criteria

Studies considered to be potentially eligible for the present meta-analysis were required to meet the following criteria: (1) original prospective or retrospective clinical studies; (2) the diseases were diagnosed based on histopathology examination; (3) the samples for mutational analysis was obtained from either biopsy or surgical tumor tissue specimens or blood samples; (4) the study had employed DNA detection techniques for profiling of TP53 and targetable oncogenic mutations; (5) NSCLC patients were treated with targeted therapy regardless of line of treatment; (6) compared progression-free survival and/or overall survival for TP53 mutation versus TP53 non-mutation subgroups (7) Hazard ratios (HRs) with 95% confidence intervals (CIs) for survival outcomes were provided directly or could be calculated from Kaplan–Meier curve alternatively, or adequate data for their estimation were provided. (8) the publication language was confined to English. Studies failing to meet these inclusion criteria were excluded.

### Data extraction

Two investigators (Kang Qin and Helei Hou) reviewed eligible studies, if there was any disagreement between the researchers, the third reviewer---Xiaochun Zhang was consulted until a consensus was reached. For each eligible study, basic information was extracted into a standardized data collection form: name of first author; year of publication; country where the study was conducted; number of patients with concurrent mutations of both TP53 and targetable EGFR/ ALK mutations and received targeted therapy; number of patients in TP53 mutation and TP53 non-mutation subgroups; methods of gene detection; specimen type (tissue or blood sample); tumor histology; stage of disease (tumor grades); targeted drugs; line of treatment; outcome of study; line of treatment; analysis method for HRs; outcomes of patients.

Data for PFS and OS were extracted as the hazard ratios (HRs) and its 95% confidence interval (CI). If the PFS and OS were evaluated by both multivariate analysis and univariate analysis, the HR and corresponding 95% CI analyzed by multivariate model were used. If the HR and its variance were not available directly, we calculated these values with raw data such as *P*-values of the log-rank test, total deaths/recurrences and total cases/controls, which were provided directly or could be calculated by reading Kaplan-Meier (KM) curve in the original articles. In addition, we used Engauge Digitizer 10.0 software (http://markummitchell.github.io/engauge-digitizer/) to extract the survival data from a Kaplan-Meier curve in some articles. The corresponding author was contacted to provide additional information if necessary.

### Methodological quality assessment

We evaluated the quality of each study included with the Newcastle-Ottawa quality assessment scale in which assessing factors included selection, comparability, and exposure [36]. The maximum NOS score is 9 points and the quality is assessed by the score as follows: ≥7, high quality; ≥5 to < 7, medium quality; < 5, low quality.

### Sensitivity analysis and publication bias assessment

Sensitivity analyses were performed by omitting each of the individual study (when there were three or more studies in comparison). Begg’s funnel plot [37] and Egger’s test [38] were applied to detect publication bias through STATA 12.0 software. The heterogeneity *P*-value < 0.05 was considered as statistically significant.

### Overall statistical analysis

The primary outcomes for this meta-analysis were PFS and OS. PFS was defined as the time from start of first-generation TKIs (gefitinib/erlotinib/icotinib) treatment until disease progression or death (whichever occurred first): OS was defined as the time from the commencement of TKIs until death of any cause. HRs for PFS and OS with 95% CIs were used to investigate the association between TP53 mutational status and response to targeted therapy in patients with advanced NSCLC. The statistical heterogeneity across the studies was evaluated through a forest plot, the Chi-square-based Q statistical test and the inconsistency statistic (I^2^) [39].

A *p*-value less than 0.10 for the Q-test indicated the existence of heterogeneity among the included studies. According to the Cochrane systematic reviews, an I^2^ lower than 25%, indicates little or no heterogeneity; 25–50% indicates moderate heterogeneity; 50–75% indicates considerable heterogeneity.

We used the random-effects model to calculate the pooled HR in case of potential heterogeneity across the studies. Subgroup analysis and sensitivity analysis were performed to evaluate the sources of heterogeneity. Begg’s funnel plots and Egger’s tests were performed to evaluate potential publication bias. A calculated HR value > 1 suggested a shorter survival for those with TP53 mutations. All CIs had a two-sided probability coverage of 95%. The 95% CI of HRs crossing 1 indicated that the correlation between TP53 mutation and prognosis was not statistically significant. *P* values for all comparisons were two-tailed, and a *p* < 0.05 indicated statistical significance for all tests except for those comparisons for heterogeneity.

## Results

### Study identification and selection

**Fig. 1:**
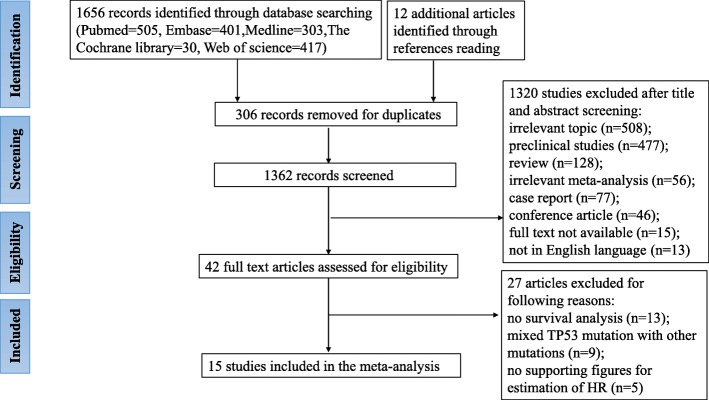
Flow diagram of included studies for this meta-analysis

Detailed process of the performed literature searches was illustrated in a flow chart (Fig. 1). A total of 1656 relevant articles were identified initially through database searching from Pubmed, Embase, Medline, The Cochrane library, and Web of science up to May 15, 2019. Additional 12 articles were identified through references reading. 306 articles were excluded for duplicates. Following a title/abstract screening and a full text reviewing by all the authors, 15 studies [25–28, 33, 34, 40–48] were included for final analysis.

### Characteristics of the included studies

The characteristics of the 15 studies were summarized in Table 1. The studies were carried out in 7 different countries (including USA, Italy, Canada, Germany, UK, China, and Korea) and were published between the year 2013 to 2019. In total, 3786 lung cancer patients who accepted genomic profiling for one or more targeted gene were included in our study. Among all patients, 1342 patients were diagnostic as EGFR- or ALK-mutated advanced NSCLC (IIIB/IV/recurrent stage) and were treated with standardized EGFR-TKIs or ALK-TKIs targeted therapy. The technique of gene detection in most studies (11/15) was next generation sequencing (NGS) with which full genome profiling were conducted. Among all, 14 studies used tissue samples for gene detection [25–28, 33, 34, 41–48], except for study by Tsui DWY et al. [40] in which blood samples were collected for gene detection. With respect to type of study, study by Aisner et al. [25] and Tsui DWY et al. [40] were performed prospectively, and study by Christopoulos P [41] were designed as both prospective and retrospective study, all the remaining 13 studies were retrospective studies. As for the survival outcomes, 15 eligible studies were divided into 3 datasets: 3 for PFS [41, 46, 47], 3 for OS [25–27], 9 for both PFS and OS.

**Table 1:** Characteristics of the included studies for the meta analyses

Study	Year	Country	N (1)	N (2)	N (3)	Percent of TP53 co-mutation	Methods of gene detection	Sample	Clinical stage	Pathological type	Detected exons of TP53	Study type
CLCGP	2013	USA	1255	80	27/53	33.75%	Sanger Sequencing	tissue	III, IV	NSCLC	2–11	Retro
Molina-Vila	2014	Europe	193	193	50/143	25.91%	HRM technique	tissue	IIIB, IV	NSCLC	5–8	Retro
Bria E	2015	Italy	18	18	7/11	38.89%	NGS or Sanger sequencing	tissue	IIIB, IV, recurrent	ADC	2–11	Retro
Canale M	2017	Italy	123	123	37/86	30.08%	Direct Sequencing	tissue	advanced	ADC (98.5%)	5–8	Retro
Labbé C	2017	Canada	105	60	24/36	40%	NGS or Sanger sequencing	tissue	advanced	ADC (96.19%)	5–10/2–11	Retro
Vander Laan	2017	USA	171	20	10/10	50%	Sanger sequencing	tissue	IV, recurrent	ADC	5–8	Retro
Aisner	2018	USA	904	35	21/14	60%	NGS	tissue	IV, recurrent	NSCLC	2–11	Pro
Tsui DWY	2018	UK	50	30	12/18	40%	TAm-Seq	plasma	IV	NSCLC	5–8	Pro
HelenaA.Yu	2018	USA	200	200	119/81	59.5%	NGS	tissue	IV	ADC	2–11	Retro
Youjin Kim	2018	Korea	200	75	43/32	57.33%	NGS	tissue	advanced	NSCLC	2–11	Retro
Youjin Kim	2018	Korea	–	82	50/32	60.98%	NGS	tissue	advanced	NSCLC	2–11	Retro
Rachiglio AM	2019	China	133	133	23/110	17.29%	NGS	tissue	advanced, metastatic	NSCLC	2–11	Retro
Kron A	2018	Germany	216	93	22/71	26.19%	NGS	tissue	IIIB, IV	ADC	2–11	Retro
Kron A	2018	Germany	–	33	11/22	33.33%	NGS	tissue	IIIB, IV	ADC	2–11	Retro
Yu Y	2018	China	52	35	21/14	60%	Illumina HiSeq400 platform	tissue	III, IV	ADC	2–11	Retro
Christopoulos P	2019	Germany	102	68	21/47	30.88%	NGS	tissue	IV	ADC	2–11	Mixed
Song P	2019	China	64	64	15/49	23.44%	NGS	tissue	IIIB, IV	ADC (98.4%)	2–11	Retro
Study		Targeted drugs			Treatment line		Outcome		Analysis method		HR estimation	
CLCGP		EGFR-TKIs			All lines		OS		Multivariate		Calculated from raw data	
Molina-Vila		EGFR-TKIs			All lines		OS		Multivariate		Reported	
Bria E		EGFR-TKIs			First line		PFS, OS		Univariate		Reported	
Canale M		EGFR-TKIs			First line		PFS, OS		Univariate		Reported	
Labbé C		EGFR-TKIs			First line		PFS, OS		Multivariate		Reported	
Vander Laan		EGFR-TKIs			All lines		PFS, OS		Multivariate		SC	
Aisner		EGFR-TKIs			All lines		OS		Not reported		SC	
Tsui DWY		EGFR-TKIs			First line		PFS, OS		Not reported		Reported	
HelenaA.Yu		EGFR-TKIs			All lines		PFS, OS		Not reported		Calculated from raw data	
Youjin Kim		EGFR-TKIs^a^			First line		PFS, OS		Multivariate		Reported	
Youjin Kim		EGFR-TKIs^b^			second line		PFS, OS		Multivariate		Reported	
Rachiglio AM		EGFR-TKIs			First line		PFS, OS		Multivariate		Reported	
Kron A		ALK-TKIs^c^			All lines		PFS, OS		Multivariate		Reported	
Kron A		ALK-TKIs^d^			All lines		PFS, OS		Multivariate		Reported	
Yu Y		ALK-TKIs			First line		PFS		Not reported		Reported	
Christopoulos P		ALK-TKIs			All lines		PFS		Not reported		Calculated from raw data	
Song P		ALK-TKIs			All lines		PFS		univariate		Reported	

N (1), number of patients involved in each study; N (2), number of patients with concurrent mutations of both TP53 and targetable EGFR or ALK mutations and received targeted therapy; N3, number of patients with/without concurrent TP53 mutations; ^a^, first and second generation EGFR-TKIs---gefitinib, erlotinib, afatinib; ^b^, third generation EGFR-TKIs---osimertinib, olmutinib; ^c^, first generation ALK-TKIs--- crizotinib; ^d^, ceritinib, alectinib, second generation ALK-TKIs---brigatinib

*Abbreviations: NGS* Next-generation sequencing, *TAm-Seq* Tagged-amplicon deep sequencing, *Retro* Retrospective study, *Pro* Prospective study, *EGFR* Epidermal growth factor receptor, *ALK* Anaplastic lymphoma kinase, *TKI* Tyrosine kinase inhibitor

All 1342 patients included were stratified according to TP53 mutation status. Totally, 475 patients were TP53-positive cases and 867 were TP53-wild type cases. Among all patients included, 1049 in 11 studies [25–28, 33, 34, 40–48] harbored EGFR active mutations (mainly EGFR Exon19 deletions and Exon 21 L858R mutations) and received EGFR-TKIs therapy (first generation EGFR-TKIs---gefitinib, erlotinib; second generation EGFR-TKIs---afatinib, dacomitinib; third generation EGFR-TKIs---osimertinib, olmutinib). Four studies with 293 patients investigated the impact of TP53 mutational status on outcome of patients with activating ALK rearrangements (mainly EML4-ALK fusions) receiving ALK-TKIs therapy (first generation ALK-TKIs----crizotinib; next generation ALK-TKIs---ceritinib, alectinib, brigatinib, ect), percent of TP53 concurrent mutations in ALK-rearranged advanced NSCLC in these four studies ranged from 23.44–60%. All these 293 patients were lung adenocarcinoma patients with ALK-rearrangement and were treated with ALK-TKIs in all lines setting (postoperative adjuvant treatment, first line treatment, second line treatment and other conditions) [41, 45–47]. Driver gene alterations and targeted drugs in the studies included were shown in detail in Table 2.

**Table 2:** Targeted gene alterations and drugs of the included studies for the meta analyses

Study	Targeted gene alteration	Detailed genotypes	Targeted drugs
CLCGP	EGFR mutations	Not shown	gefitinib, erlotinib
Molina-Vila	EGFR mutations	Not shown	erlotinib
Bria E	EGFR mutation	Exon 19 deletion; Exon 21 L858R mutation	gefitinib
Canale M	EGFR mutation	Exon 19 deletion Exon 21 L858R mutations Exon 21 L861Q mutations Exon 18 mutations	gefitinib, erlotinib, afatinib, dacomitinib
Labbé C	EGFR mutation	Exon 19 deletion Exon 21 L858R mutation Exon 18 mutations, Exon 19 insertion, Exon 19 L747P mutation Multiple mutations (Exons 21 + 20, exons 19 + 20, exons 21 + 18)	gefitinib, erlotinib
Vander Laan	EGFR mutation	Exon 19 deletions Exon 21 L858R mutation Exon 18 G719X mutation Exon 20 insertions Exon 20 S768I mutation	gefitinib, erlotinib, afatinib
Aisner	EGFR mutation	Exon 19 deletions Exon 21 L858R mutation Exon 18 G719X mutation Exon 21 L861Q mutation	EGFR-TKIs
Tsui DWY	EGFR mutation	EGFR Exon 21 L858R mutations	gefitinib
HelenaA.Yu	EGFR mutation	EGFR T790M mutation Exon 19 deletion Exon21 L858R mutation Exon 20 insertions Exon 18 deletion Exon 19 insertion Exon21 L861Q mutation Exon 18 G719X mutation Exon 19 L747P. mutation Exon 18 E709X mutation + Exon 18 G719X mutation Exon 18 G719X+ Exon 20 S768I mutation Exon 18 G719X+ Exon21 L861Q mutation	erlotinib
Youjin Kim	EGFR mutation	Exon21 L861Q mutation Exon 19 deletion Exon21 L858R mutation Exon18 G719A mutation Exon21 L833V mutation + Exon21 L858R mutation Exon21 L833V+ Exon21 H835L mutation	gefitinib, erlotinib, afatinib, osimertinib, olmutinib
Rachiglio AM	EGFR mutation	Exon 19 deletion Exon21 L858R Exon20 T790M	gefitinib, erlotinib, afatinib,
Kron A	ALK re-arrangements	EML4-ALK fusions	crizotinib, ceritinib, alectinib, brigatinib
Yu Y	ALK re-arrangements	EML4-ALK fusions Non-EML4-ALK fusions	crizotinib
Christopoulos P	ALK re-arrangements	EML4-ALK fusions KIF5B-ALK fusions	ALK-TKIs
Song P	ALK re-arrangements	EML4-ALK fusions Non-EML4-ALK fusions	ALK-TKIs

*Abbreviations: EGFR* Epidermal growth factor receptor, *ALK* Anaplastic lymphoma kinase, *TKI* Tyrosine kinase inhibitor

Percent of TP53 concurrent mutations in EGFR-mutated advanced NSCLC in these 11 studies ranged from 25.91–60%. In terms of the pathology type of tumor, 9 studies focused on ADC only or over 96% of patients included were ADC patients [28, 33, 38, 43, 48]; the remaining 6 studies included patients with all NSCLC types (adenocarcinoma, squamous carcinoma, adeno-squamous carcinoma, neuroendocrine carcinoma, poorly differentiated carcinoma, ect) [25–27, 34, 40, 43]. When it comes to treatment lines, 379 out of 1049 patients in 5 studies received first line treatment of first- or second-generation EGFR-TKIs [26, 34, 40, 42, 43]; the remaining 670 patients received EGFR-TKIs treatment in all lines setting (postoperative adjuvant treatment, first line treatment, second line treatment and other conditions).

Notably, patients in study by Youjin Kim et al. [43] were divided in two independent groups according to different EGFR TKIs treatments: group1, 1st/2nd-generation EGFR TKIs (first-line treatment); group 2, 3rd-generation EGFR-TKIs following initial EGFR-TKIs failure (second-line treatment). Similarly, patients in study by Kron A et al. [45] were also grouped based on types of ALK-TKIs therapy-patients with first generation ALK-TKIs or next generation ALK-TKIs (ceritinib/alectinib/brigatinib). Prognosis and clinical outcomes of patients in different groups in these two studies were presented independently. Therefore, survival information (PFS/OS with HR and 95%CI) of patients in these two studies were extracted separately accordingly, as shown in Table 1.

### Methodological quality assessment

The Newcastle-Ottawa scale scores of the 15 eligible studies were all above 6 (7.73.

on average), indicating high quality of study (Table 3).

**Table 3:** Quality assessment of eligible studies using the Newcastle Ottawa quality assessment scale

Study	Selection^a^	Comparability^b^	Outcome^c^	Total (quality) score^d^	Quality	Ref
CLCGP	4	2	2	8	High	[26]
Molina-Vila	4	1	2	7	High	[27]
Bria E	3	2	3	8	High	[42]
Canale M	2	2	3	7	High	[44]
Labbé C	3	2	3	8	High	[33]
VanderLaan	3	1	2	6	Medium	[28]
Aisner	3	2	3	8	High	[25]
Tsui DWY	3	2	2	7	High	[40]
Helena A.Yu	3	2	3	8	High	[48]
Kim Y	4	2	3	9	High	[43]
Rachiglio AM	4	2	2	8	High	[34]
Kron A	4	2	1	7	High	[45]
Yu Y	3	2	3	8	High	[46]
Christopoulos P	3	2	3	8	High	[41]
Song P	4	2	3	9	High	[47]

Selection^a^, graded based on 4 items as follows: firstly, representativeness of the exposed cohort (0 points, selected group of users, or no description of the derivation of the cohort;1 point, truly or somewhat representative of the average level in the community); secondly, selection of the non-exposed cohort (0 point, drawn from a different source or no description of the derivation of the non-exposed cohort; 1 point, drawn from the same community as the exposed cohort); thirdly, ascertainment of exposure (0 point, written self-report or no description; 1 point, secure record or structured interview); fourthly, demonstration that outcome of interest was not present at the start of the study (0 point, no;1 point, yes). Comparability^b^, graded as 0–2 points (0 point, study controls without the most important factor or any additional factor; 1 point, study controls for the most important factor or any additional factor; 0 points, study controls for the most important factor and any additional factor). Outcome^c^, graded based on 3 items: firstly, assessment of outcome (1point, independent blind assessment or record linkage; 0 point, self-report or no description); secondly, was follow-up long enough for outcomes to occur? (1 point, yes; 0 point, no); thirdly, adequacy of follow-up of cohorts (1point, complete follow-up or subjects lost to follow-up unlikely to introduce bias; 0 point, follow-up rate < 80% and no description of those lost, or no statement)

### Overall analysis

**Fig. 2:**
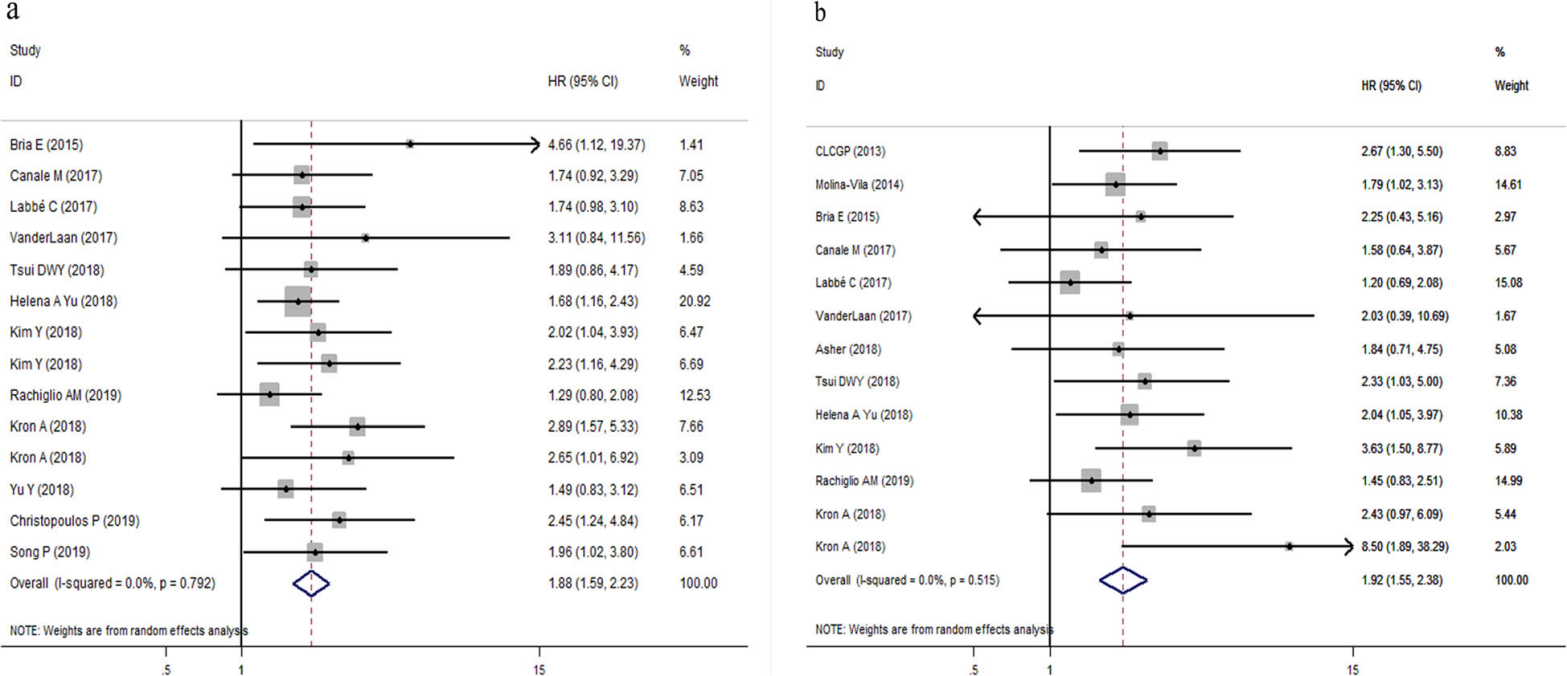
Forest plots of pooled HRs of overall PFS and OS between the wild type and the TP53 mutation patients with advanced NSCLC (**a**) PFS; (**b**) OS. *Abbreviations*: *HR* hazard ratio, *TP53* tumor protein p53, *PFS* progression-free survival, *OS* overall survival, *NSCLC* non-small cell lung cancer

Taken together, when compared with the TP53 mutation group, the wild-type group was associated with significantly longer PFS (HR,1.88, 95% CI: 1.59–2.23, *P* < 0.001) (Fig. 2a) and longer OS (HR = 1.92, 95% CI: 1.55–2.38, *P* < 0.001) (Fig. 2b) values. No heterogeneity was observed among the included studies regarding either PFS(I^2^ = 0.0%, *P* = 0.792) or OS (I^2^ = 0.0%, *P* = 0.515). Generally, these findings showed that TP53 mutations were associated with reduced responsiveness and poor prognosis of patients with advanced NSCLC who received targeted therapy of EGFR-TKIs or ALK-TKIs.

### Subgroup analysis

**Fig. 3:**
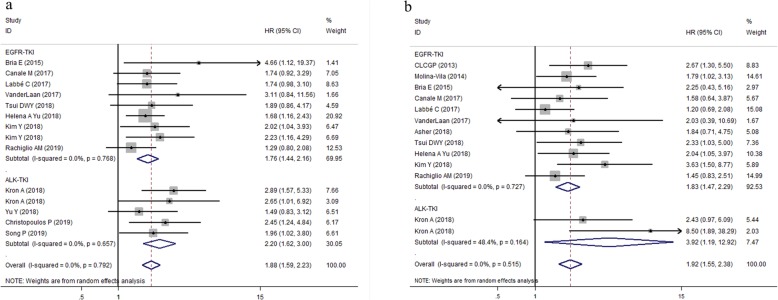
Forest plots of pooled HRs of PFS and OS between the wild type and the TP53 mutation patients based on TKIs therapy (**a**) PFS; (**b**) OS. *Abbreviations*:* HR* hazard ratio, *PFS* progression-free survival, *OS* overall survival, *TP53* tumor protein 53, *TKI* tyrosine kinase inhibitor, *EGFR* epidermal growth factor receptor, *ALK* anaplastic lymphoma kinase

**Fig. 4:**
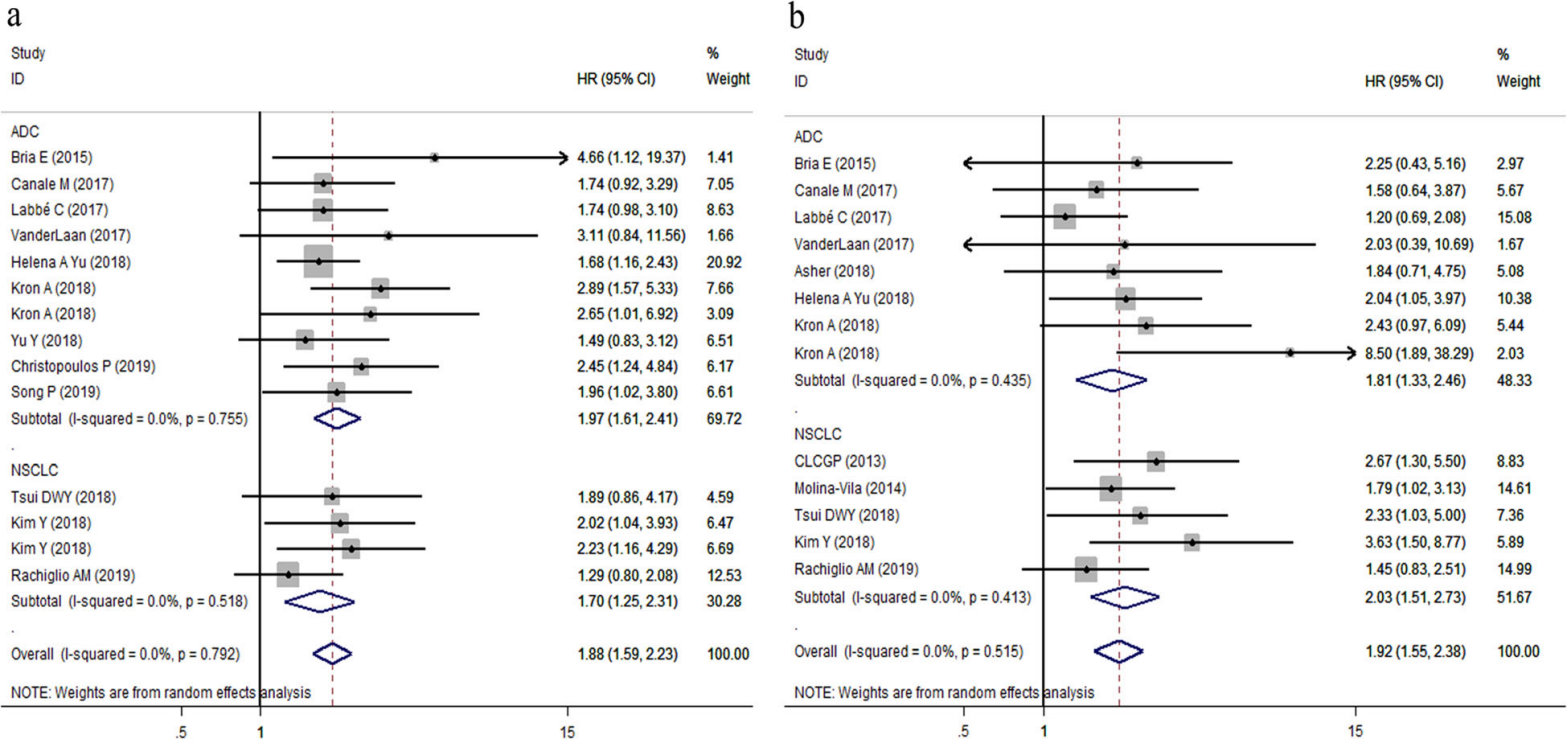
Forest plots of pooled HRs of PFS and OS between the wild type and the TP53 mutation patients based on histopathological type of tumor (ADC or all NSCLC subtypes (**a**) PFS; (**b**) OS. *Abbreviations*: *HR* hazard ratio, *PFS* progression-free survival, *OS* overall survival, *TP53* tumor protein 53, *TKI* tyrosine kinase inhibitor, *ALK* anaplastic lymphoma kinase, *ADC* adenocarcinoma, *NSCLC* non-small cell lung cancer

**Fig. 5:**
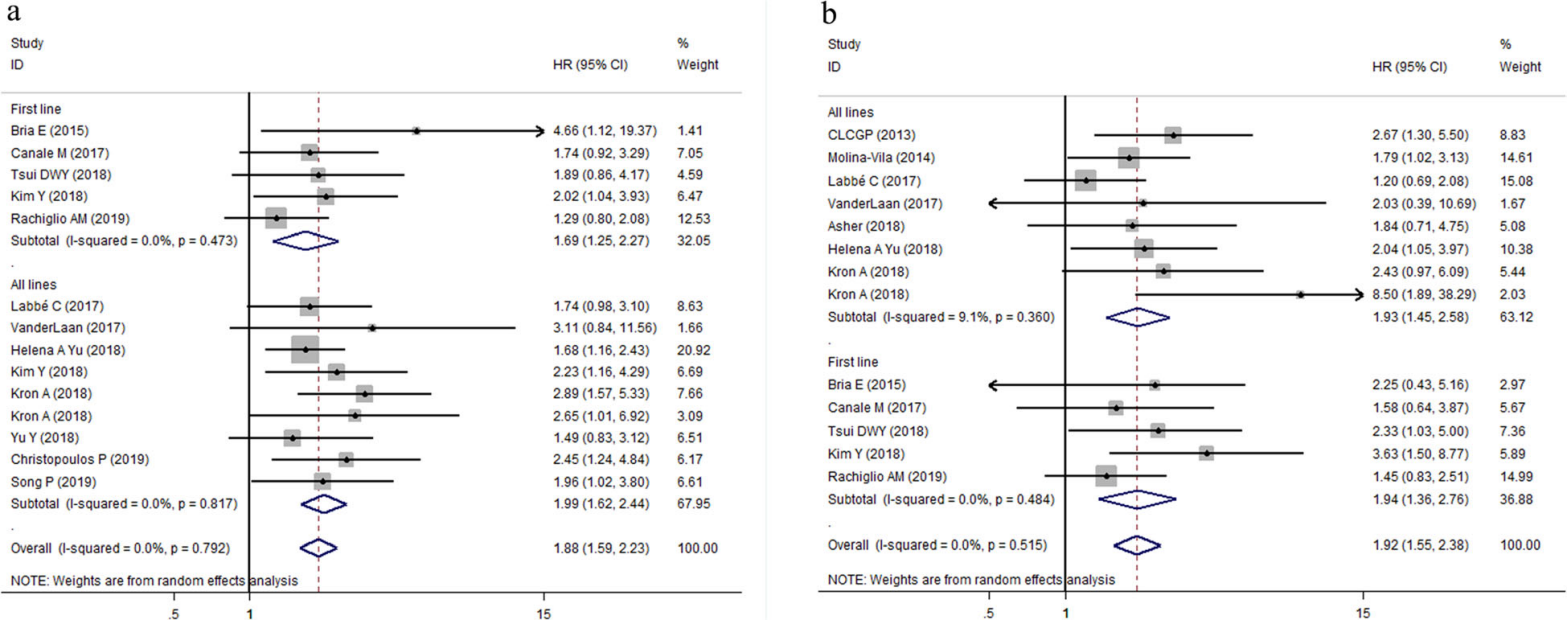
Forest plots of pooled HRs of PFS and OS between the wild type and the TP53 mutation patients based on line of treatment (first line or all lines) (**a**) PFS; (**b**) OS. *Abbreviations*: *HR* hazard ratio, *PFS* progression-free survival, *OS* overall survival, *TP53* tumor protein 53

To comprehensively explore the association between TP53 mutation and survival of patients with EGFR- TKIs or ALK-TKIs treatments, subgroup analyses were performed based on type of targeted drugs (EGFR-TKIs or ALK-TKIs) (Fig. 3a,b); histopathological type of tumor (ADC or all NSCLC subtypes) (Fig. 4a,b); treatment line of targeted therapy (first line or all lines) (Fig. 5a,b). For patients with EGFR-TKIs therapy in 11 studies, TP53 mutations were associated with higher risk of disease progression (pooled HR for PFS: 1.76, 95% CI: 1.44–2.16, *P* < 0.001; heterogeneity: I^2^ = 0.0%, *P* = 0.768), and death (pooled HR for OS:1.83, 95% CI: 1.47–2.29, *P* < 0.001; heterogeneity: I^2^ = 0.0%, *P* = 0.727) (Fig. 3 a,b). For 293 patients who harbored ALK oncogenic mutations and underwent ALK-TKIs therapy, the pooled HR for PFS was 2.20 (95% CI: 1.62–3.00, *P* < 0.001; heterogeneity: I^2^ = 0.0%, *P* = 0.657) **(**Fig. 3a); data concerning the overall survival in patients with ALK-TKIs therapy was only available in study by Kron A et al., in which 93 patients were under treatment with first-generation ALK inhibitors and 33 patients were under treatment with next-generation ALK inhibitors. Pooled HR for OS of these two groups of patients was 3.92 (95%CI: 1.19–12.92, *P* = 0.025; heterogeneity: I^2^ = 48.4%, *P* = 0.164) (Fig. 3b).

With regard to type of tumor, concurrent TP53 mutations were poor prognostic factors in patients with both ADC (Pooled HR for PFS = 1.97, 95% CI:1.61–2.41, *P* < 0.001, heterogeneity: I^2^ = 0.0%, *P* = 0.755; Pooled HR for OS = 1.81, 95% CI: 1.33–2.46, *P* < 0.001, heterogeneity: I^2^ = 0.0%, *P* = 0.435) and all NSCLC subtypes (Pooled HR for PFS = 1.70, 95% CI:1.25–2.31, *P* = 0.001, heterogeneity: I^2^ = 0.0%, *P* = 0.518; Pooled HR for OS = 2.03, 95% CI: 1.51–2.73, *P* < 0.001, heterogeneity: I^2^ = 0.0%, *P* = 0.413) (Fig. 4a,b). When the analysis was restricted to line of targeted therapy, TP53 mutations were associated with reduced PFS and OS in patients with both first-line targeted therapy (pooled HR for PFS: 1.69, 95% CI: 1.25–2.27, *P* = 0.001, heterogeneity: I^2^ = 0.0%, *P* = 0.473; pooled HR for OS: 1.94, 95% CI: 1.36–2.76, *P* < 0.001, heterogeneity: I^2^ = 0.0%, *P* = 0.484) (Fig. 5a) and those who received EGFR-TKIs treatments in all lines setting (pooled HR for PFS: 1.99, 95% CI: 1.62–2.44, *P* = 0.001, heterogeneity: I^2^ = 0.0%, *P* = 0.817; pooled HR for OS: 1.93, 95% CI: 1.45–2.58, *P* < 0.001, heterogeneity: I^2^ = 9.1%, *P* = 0.360) (Fig. 5b).

**Fig. 6:**
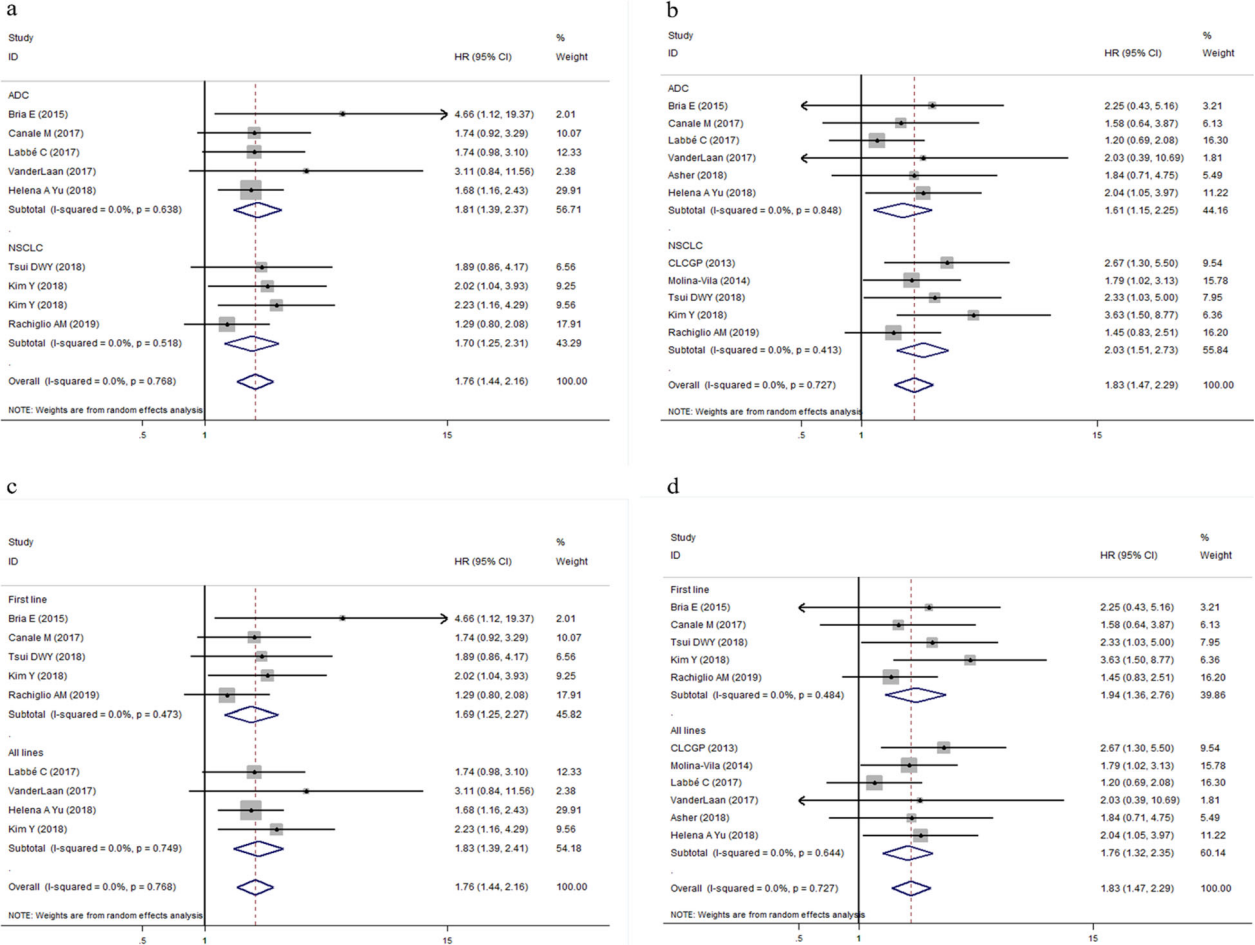
Forest plots of pooled HRs of PFS and OS between the wild type and the TP53 mutation in patients with EGFR-TKIs treatment (**a**) subgroup analysis for pooled HR of PFS based on pathological type (**b**) subgroup analysis for pooled HR of OS based on pathological type. *Abbreviations*: *HR* hazard ratio, *PFS* progression-free survival, *OS* overall survival, *EGFR* epidermal growth factor receptor, *TKI* tyrosine kinase inhibitor, *ADC* adenocarcinoma, *NSCLC* non-small cell lung cancer

Since all patients included in our study who received ALK-TKIs therapy were diagnosed as ADCs and received ALK-TKIs as all line treatments, so we further performed subgroup analysis in patients with EGFR-TKIs therapy based on pathological type of cancer, and treatment line. In the subgroup of patients with adenocarcinoma, the pooled HR for PFS was 1.81 (95% CI: 1.39–2.37, *P* < 0.001; heterogeneity: I^2^ = 0.0%, *P* = 0.638); the pooled HR for OS was 1.61 (95%CI: 1.15–2.25, *P* < 0.001; heterogeneity: I^2^ = 0.0%, *P* = 0.848). In addition, for those 5 studies investigating the impact of TP53 mutational status on clinical efficacy of EGFR-TKIs in all NSCLCs generally, the pooled HR for PFS (HR = 1.70, 95% CI 1.25–2.31, *P* < 0.001; heterogeneity: I^2^ = 0.0%, *P* = 0.518); the pooled HR for OS (HR =2.03, 95% CI 1.51–2.73, *P* < 0.001; heterogeneity: I^2^ = 0.0%, *P* = 0.413) (Fig. 6 a, b).

When stratifying patients according to treatment line, the observed results indicated that TP53 mutations were associated with a significantly reduced PFS and OS in patients who received EGFR-TKIs treatment in the first-line setting and those who received EGFR-TKIs treatment in all lines generally. For patients who received first/second generation of EGFR-TKIs as first line therapy, the pooled HR for PFS (HR = 1.69, 95% CI 1.25–2.27, *P* < 0.001; heterogeneity: I^2^ = 0.0%, *P* = 0.473), and the pooled HR for OS (HR =1.94, 95% CI 1.36–2.76, *P* < 0.001; heterogeneity: I^2^ = 0.0%, *P* = 0.484); In the remaining 6 studies which included NSCLC patients treated with EGFR-TKIs therapy regardless of the treatment lines (first line/second line/ postoperative treatments), the pooled HR for PFS (HR = 1.83, 95% CI 1.39–2.41, *P* < 0.001; heterogeneity: I^2^ = 0.0%, *P* = 0.749), The pooled HR for OS (HR = 1.76, 95% CI 1.32–2.35, *P* < 0.001; heterogeneity: I^2^ = 0.0%, *P* = 0.644) (Fig. 6 c, d).

These findings suggested that the concurrent mutations of TP53 predict a poor prognosis as well as increased risk of disease progression and death in patients with advanced NSCLC undergoing targeted TKIs therapy in all subgroups that we listed.

### Sensitivity analysis

Sensitivity analysis demonstrated that the heterogeneity and overall effect were not significantly altered by omitting any study in all groups mentioned above. (Additional file 1-11). Thus, a negative correlation between mutated TP53 and the prognosis of patients with advanced NSCLC undergoing EGFR-TKIs or ALK-TKIs therapy existed after any study was excluded from our meta-analysis.

### Publication bias

**Fig. 7:**
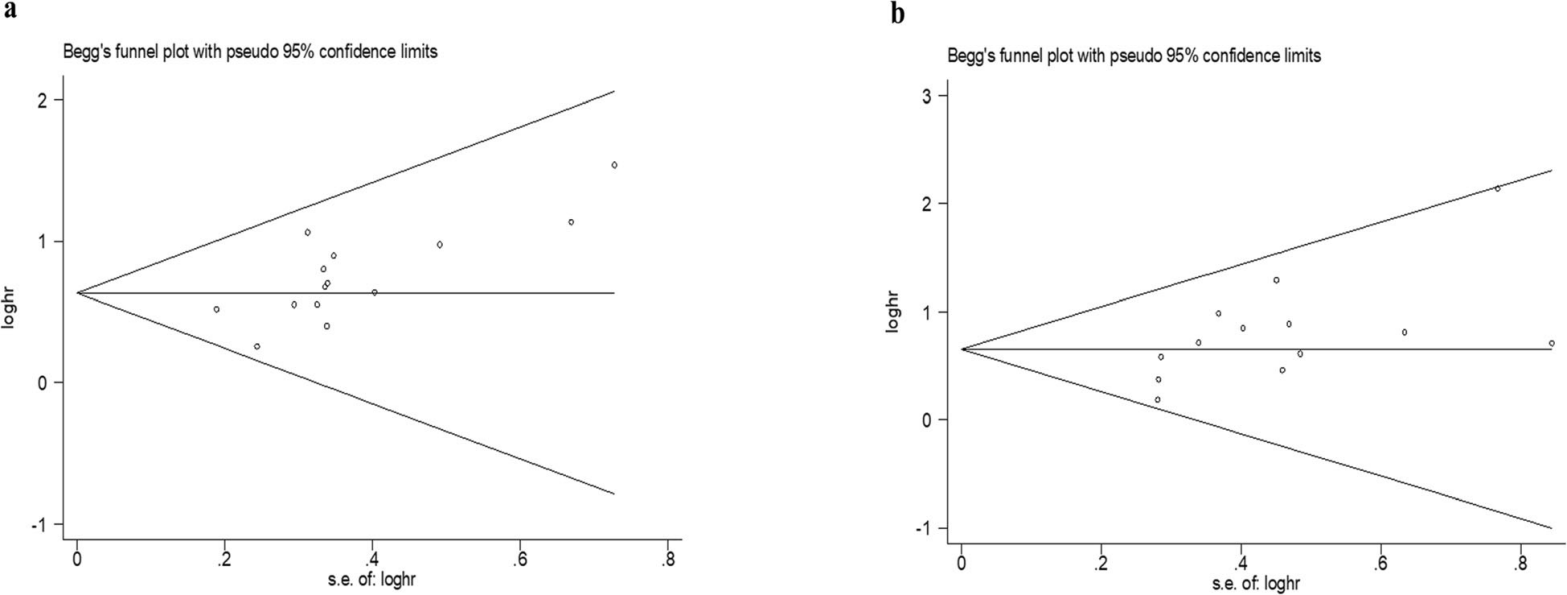
Begg’s funnel plots for publication bias of (**a**) overall PFS (**b**) overall OS. *Abbreviations*: *PFS* progression-free survival, *OS* overall survival *Abbreviations*: *PFS* progression-free survival, *OS* overall survival

The Begg’s funnel plots revealed an almost symmetrical distribution in terms of overall PFS and overall OS of the 15 studies included in this meta-analysis (Fig. 7a,b), and Egger’s tests indicated no evidence of publication bias (overall PFS, *P* = 0.057; overall OS, *P* = 0.077).

## Discussion

The development of targeted therapies has advanced the therapeutic strategy of NSCLC from conventional chemo-and radiation-based therapy to genetic alteration-guided targeted therapy. However, despite the excellent initial efficacy of EGFR and ALK inhibitors in patients with sensitive EGFR or ALK mutations, resistance occurs almost inevitably in both conditions. Mechanisms underlying multiple TKI resistance have not yet been fully elucidated and remain a major concern.

In our study, TP53 mutations were observed in 43.74% (423/967) of all EGFR-mutated patients and 30.72% (90/293) of all ALK-mutated NSCLC cases, which were in accordance with previous reports [49, 50]. The main function domain of TP53 is the DNA-binding domain (DBD) encoded by exon5–8, and in our meta-analysis, all the included studies for the detection of TP53 mutation covered these exons. The high frequency of these mutations can disrupt the normal function of TP53 and as a consequence, disruptions in cell cycle, DNA repair, apoptosis signaling occur, which might account for early tumor development and progression in NSCLC patients with targeted therapy.

In recent years, there has been an increasing number of studies discussing about the significance of TP53 mutations for prognosis in patients with non-small cell lung cancer, and it was suggested that the impact of TP53 mutations on clinical outcome of patients varies with different TP53 mutation subtypes. According to study by Poeta et al. [51], TP53 mutations could be classified into disruptive and non-disruptive mutations, based on the degree of disturbance of the protein structure. Disruptive mutations result in a complete, or almost complete loss of function of p53 protein, whereas non-disruptive mutations refer to conservative mutations or non-conservative mutations (excluding stop codons) outside the L2–L3 region that can retain some of the functional properties of p53 protein. Among all articles included in our meta-analysis, study by Molina-Vila et al. [27], Canale M et al. [44], Song P et al. [47] all indicated that a non-disruptive TP53 alteration had an independent, significant association with shortened survival as compared with the wild-type. However, data provided in these articles was not enough to perform a subgroup analysis based on type of TP53 mutations (disruptive or nondisruptive).

In addition, TP53 genetic mutations could be classified into missense and non-missense mutations on basis of mutations subtypes. Missense mutation refers to a mutation that results in a single amino acid change, and any genetic alterations other than missense mutations including nonsense mutations (introducing a stop codon), deletions, insertions (in-frame or producing a frame shift) and substitutions at splice sites were defined as non-missense mutations [51, 52]. Study by Labbé et al. showed that patients with TP53 missense mutations, instead of non-missense mutations, led to significantly shorter PFS for NSCLC patients with first line EGFR TKI therapy [33]. More studies are needed to confirm the prognostic impact of TP53 mutation in different types in the future.

In our study, we observed significantly shorter PFS and OS in patients with TP53 mutations than those with TP53 wild-type cases. Specifically, in patients with first line EGFR-TKIs therapy, the pooled HR for PFS was 1.69 (95% CI 1.25–2.27, *P* < 0.001; heterogeneity: I^2^ = 0.0%, *P* = 0.473), the pooled HR for OS was 1.90 (95% CI 1.42–2.54, *P* < 0.001; heterogeneity: I^2^ = 0.0%, *P* = 0.678), indicating that TP53 concurrent mutations.

might be involved in primary resistance to EGFR-TKIs in patients with advanced NSCLC. Despite the well-advanced elucidation of resistance mechanisms, it remains unclear why some patients with sensitive oncogenic mutations with TP53 mutations.

relapse faster or show worse up-front response to EGFR/ALK-TKIs treatments. Indeed, preclinical studies indicated that increased Fas expression is necessary for a full p53-dependent apoptotic response following genotoxic stress in several human cell lines [53, 54]. Then it was reported that expression of p53 protein could enhance gefitinib-induced apoptosis in NSCLC cells by upregulation of FAS, and TP53 mutations could reduce sensitivity to EGFR-TKI [30, 55]. On the other hand, it was assumed that TP53 mutations occurred in the early phase of tumorigenesis could lead to chromosomal instability thereby trigger the development of multiple resistance to targeted therapy [25, 29]. For example, in study by Alidousty et al., amplifications of multiple cancer genes including MYC, CCND1, TERT, BIRC2, ORAOV1, YAP1 were observed in 24% of all patients involved in the study who have TP53 and ALK concurrent mutations [29]. On further investigation, elevated expression levels of the EML4-ALK protein and increased cell proliferation rates were observed due toMYC binding sites within the promoter region of EML4 in ALK+/TP53-mutated cells and MYC-overexpression assuming a potential MYC-dependent resistance mechanism in patients with increased MYC copy numbers, which was in line with conclusions of study by Aisner et al. [25].

Histologic transformation of NSCLC to SCLC has already been recognized as a crucial mechanism of acquired resistance to EGFR- or ALK-TKI in EGFR-mutated [48, 56] or ALK+ adenocarcinomas [57]. Co-occurring mutations of the tumor-suppressor genes TP53 and RB1 (RB transcriptional corepressor 1) could be observed in over 75% of patients with SCLC [58, 59]. Patients with EGFR/RB1/TP53-mutant lung cancers represented approximately 5% of EGFR-mutant lung cancers and were at much higher risk for SCLC transformation than those without baseline TP53 and RB1 alterations [60]. By investigating the genetic backgrounds of patients with de novo combined SCLC/NSCLC as well as those who experienced SCLC transformation from lung adenocarcinoma after TKI treatment, Lin et al. reported a high consistency in EGFR/TP53/RB1 mutations and expression patterns of p53 and Rb in these two different histologic components of SCLC, indicating that inactivation of TP53/RB1 function might be an early event in the histogenesis of synchronous and metachronous SCLC/NSCLC [61]. In EGFR-mutant lung cancers, RB1 alterations almost always occur concurrently with TP53/EGFR-mutant lung cancers with transformation mimic classical SCLC with RB1 and TP53 biallelic loss [62]. However, it has not been fully elucidated whether RB1 and TP53 loss were early events within EGFR-mutant lung cancers or were acquired late in the process of histologic shift. RB1 and TP53 loss seem necessary instead of sufficient to induce lineage plasticity [60, 62]. It is worthwhile to carry out more studies regarding this topic to further elucidate this question in the future.

Nowadays, the development of liquid biopsy allows a real-time biomolecular profiling of the tumor through the analysis of human body fluids, such as plasma, pleural effusions and urine, etc. [63]. Cell free DNA (cfDNA), which refers to the free DNA fragments in circulation (plasma or serum) derived from tumor cells, is the most widely adopted source for tumor genotyping in advanced NSCLC [64, 65]. Multiple studies have confirmed that it was reliable to identify guideline-recommended biomarkers in patients with mNSCLC through a comprehensive cfDNA test, especially with the highly sensitive and specific NGS-based detection [66, 67]. For example, in the Non-invasive versus Invasive Lung Evaluation (NILE) study, plasma NGS tests in previously untreated mNSCLC showed high sensitivity and specificity with high tissue concordance, significantly faster return of results, and was even more rapidly and completely than the standard-of-care tissue genotyping [66]. The convenience, minimal invasiveness and repeatability of liquid biopsy enable the utility of cfDNA detection as a significant way not only for the detection of targetable driver alterations, but also for exploration of mechanisms of resistance to such drugs [68, 69]. Studies by Iwama et al. confirmed the presence of de novo TP53 mutations in plasma before afatinib treatment were involved in developmet of resistance to this drug [68]; Furthermore, Christopoulos et al. recently reported that detection of TP53 mutations in tissue or liquid rebiopsies at disease progression identified ALK+ lung cancer patients with poor survival, and acquisition of TP53 mutations at progression was associated with more aggressive disease, shorter TKI responses and inferior OS in comparable to those primary TP53 mutated cases [70].With the widespread utility of NGS-based cfDNA detection in genomic variants assessment, some scholars suggested a paradigm shift in the diagnostic algorithm of advanced NSCLC, moving from the old concept, “tissue first” to a “blood first” approach [66]. However, there are still many emerging challenges. First of all, since the concentration of ctDNA is extremely low (< 1%), increasing the test sensitivity and specificity is a key point in reducing the uninformative false negative cfDNA results Secondly, cfDNA released by non-malignant cells, for example the clonal hematopoiesis (CH) phenomenon in people without liver cancer [71], could disturb analysis results of ctDNA detection and lead to false-positive results. What’s more, consensus in standard for technique selection and statistic analysis should also be made to avoid discrepancies amongst different detecting processes.

There were several limitations in our study. First, although sensitivity analysis suggested that our results were reliable, publication bias could be a concern since only articles published in English are included in our study, articles in other languages which may report negative or insignificant results were excluded. In addition, the absolute number of studies and patients included in our meta-analysis is low. Particularly, the prognostic value of TP53 concurrent mutations in patients with ALK-TKIs treatments was a quite novel issue and only a few articles discussed this topic.

To the best of our knowledge, this is the first meta-analysis to systemically investigate the prognostic impact of TP53 mutations on patients treated with targeted therapy directed against targetable alterations in advanced NSCLC. Although limited by small number of original researches included, our study suggested that co-existence of TP53 mutations with activating EGFR mutations and ALK re-arrangements predicted limited efficacy of EGFR-TKIs and ALK-TKIs based targeted therapy and poor prognosis of patients compared with those without these mutations.

The impact of genetic complexity and heterogeneity on responsiveness to EGFR-TKIs or ALK-TKIs treatments and clinical outcomes of patients with advanced NSCLC has become a hot focus of widespread concern. Co-existing alterations like amplification of ERBB2 or MET, mutations in TP53, primary EGFR T790M mutations, BRAF fusions were claimed to portend poorer outcomes in patients with EGFR-mutant NSCLC by an increasing number of studies [16, 17, 21]. Concurrent TP53 mutations with other targetable mutations were rare events: in study by Aisner et al. [25], in addition to 35 EGFR-mutant lung adenocarcinomas, TP53 mutations were also identified in 22 KRAS mutant and 11 ALK or ROS1 or RET rearranged tumors, however, prognosis information for these patients were not available.

Routine use of massively parallel sequencing enables detection of both targetable driver alterations and tumor suppressor gene and other gene alterations that have potential significance for therapy selection and function as predictive markers for the efficacy of clinical treatment. Therefore, mastering the patient’s comprehensive mutation profile will help the clinician to optimize the individualized treatment strategies for these patients.

## Conclusions

The present systematic review and meta-analysis suggested that the presence of concurrent TP53 mutations predicts a decreased clinical efficacy of EGFR-TKIs and ALK-TKIs and is a negative prognostic factor for patients with advanced NSCLC harboring EGFR sensitive mutations or ALK re-arrangements (regardless of pathological subtype and line of treatments). Specifically, in patients with first-line EGFR-TKIs treatments, TP53 concurrent mutation is associated with significantly reduced survivals. Therefore, our study provided clinical evidence that TP53 mutations might be involved in primary resistance to EGFR-TKIs. With the underlying mechanisms remaining unclear, further researches are necessary to get better understanding of this biomarker and its role in targeted therapy of NSCLC. In addition, development of targeted drugs against TP53 mutations and comprehensive gene profiling are needed to help physicians determine the best combined regimen for patients with advanced NSCLC.

## Supplementary information

**Additional file 1: Figure S1.** Sensitivity analyses for (a) overall PFS (b) overall OS. Abbreviations: PFS, progression-free survival; OS, overall survival.

**Additional file 2: Figure S2.** Sensitivity analyses for (a) PFS (b) OS of patients with EGFR-TKIs treatments. Abbreviations: PFS, progression-free survival; OS, overall survival; EGFR, epidermal growth factor receptor; TKI, tyrosine kinase inhibitor.

**Additional file 3: Figure S3.** Sensitivity analysis of PFS for patients with ALK-TKIs therapy. Abbreviations: PFS, progression-free survival; ALK, anaplastic lymphoma kinase; TKI, tyrosine kinase inhibitor (PPTX 610 kb)

**Additional file 4: Figure S4.** Sensitivity analyses of (a) PFS and (b) OS in patients with ADC Abbreviations: PFS, progression-free survival; OS, overall survival; ADC, adenocarcinoma.

**Additional file 5: Figure S5.** Sensitivity analyses of (a) PFS and (b) OS in patients with NSCLCs. Abbreviations: PFS, progression-free survival; OS, overall survival; NSCLC, non-small cell lung cancer (PPTX 871 kb)

**Additional file 6: Figure S6.** Sensitivity analyses of (a) PFS and (b) OS in patients with first line EGFR-TKIs or ALK-TKIs treatments. Abbreviations: PFS, progression-free survival; OS, overall survival; NSCLC, non-small cell lung cancer.

**Additional file 7: Figure S7.** Sensitivity analyses of (a) PFS and (b) OS in patients EGFR-TKIs or ALK-TKIs treatments in all lines setting. Abbreviations: PFS, progression-free survival; OS, overall survival; EGFR, epidermal growth factor receptor; ALK, anaplastic lymphoma kinase; TKI, tyrosine kinase inhibitor.

**Additional file 8: Figure S8.** Sensitivity analyses of (a) PFS and (b) OS in ADC patients with EGFR-TKIs treatments. Abbreviations: PFS, progression-free survival; OS, overall survival; ADC, adenocarcinoma; EGFR, epidermal growth factor receptor; TKI, tyrosine kinase inhibitor.

**Additional file 9: Figure S9.** Sensitivity analyses of (a) PFS and (b) OS in NSCLC patients with EGFR-TKIs treatments. Abbreviations: PFS, progression-free survival; OS, overall survival; NSCLC, non-small cell lung cancer; EGFR, epidermal growth factor receptor; TKI, tyrosine kinase inhibitor.

**Additional file 10: Figure S10.** Sensitivity analyses of (a) PFS and (b) OS in patients with first line EGFR-TKIs treatments. Abbreviations: PFS, progression-free survival; OS, overall survival; EGFR, epidermal growth factor receptor; TKI, tyrosine kinase inhibitor.

**Additional file 11: Figure S11.** Sensitivity analyses of (a) PFS and (b) OS in patients with EGFR-TKIs treatments in all-lines setting. Abbreviations: PFS, progression-free survival; OS, overall survival; EGFR, epidermal growth factor receptor; TKI, tyrosine kinase inhibitor.

## Data Availability

The data sets used and analyzed in the present study are available from the corresponding author upon reasonable request.

## Abbreviations

HR: Hazard ratio
PFS: Progression-free survival
OS: Overall survival
TP53: Tumor protein 53
TKI: Tyrosine kinase inhibitor
EGFR: Epidermal growth factor receptor
ALK: Anaplastic lymphoma kinase
ADC: Adenocarcinoma
NSCLC: Non-small cell lung cancer
NGS: Next-generation sequencing
TAm-Seq: Tagged-amplicon deep sequencing
Retro: Retrospective study
Pro: Prospective study
