# Supplementary figures and images for: Prognostic value of TP53 concurrent mutations for EGFR- TKIs and ALK-TKIs based targeted therapy in advanced non-small cell lung cancer: a meta-analysis
Source: BMC Cancer. 2020 Apr 16;20:328. doi: 10.1186/s12885-020-06805-5 (PMC7164297; doi:10.1186/s12885-020-06805-5)

## Slide 1
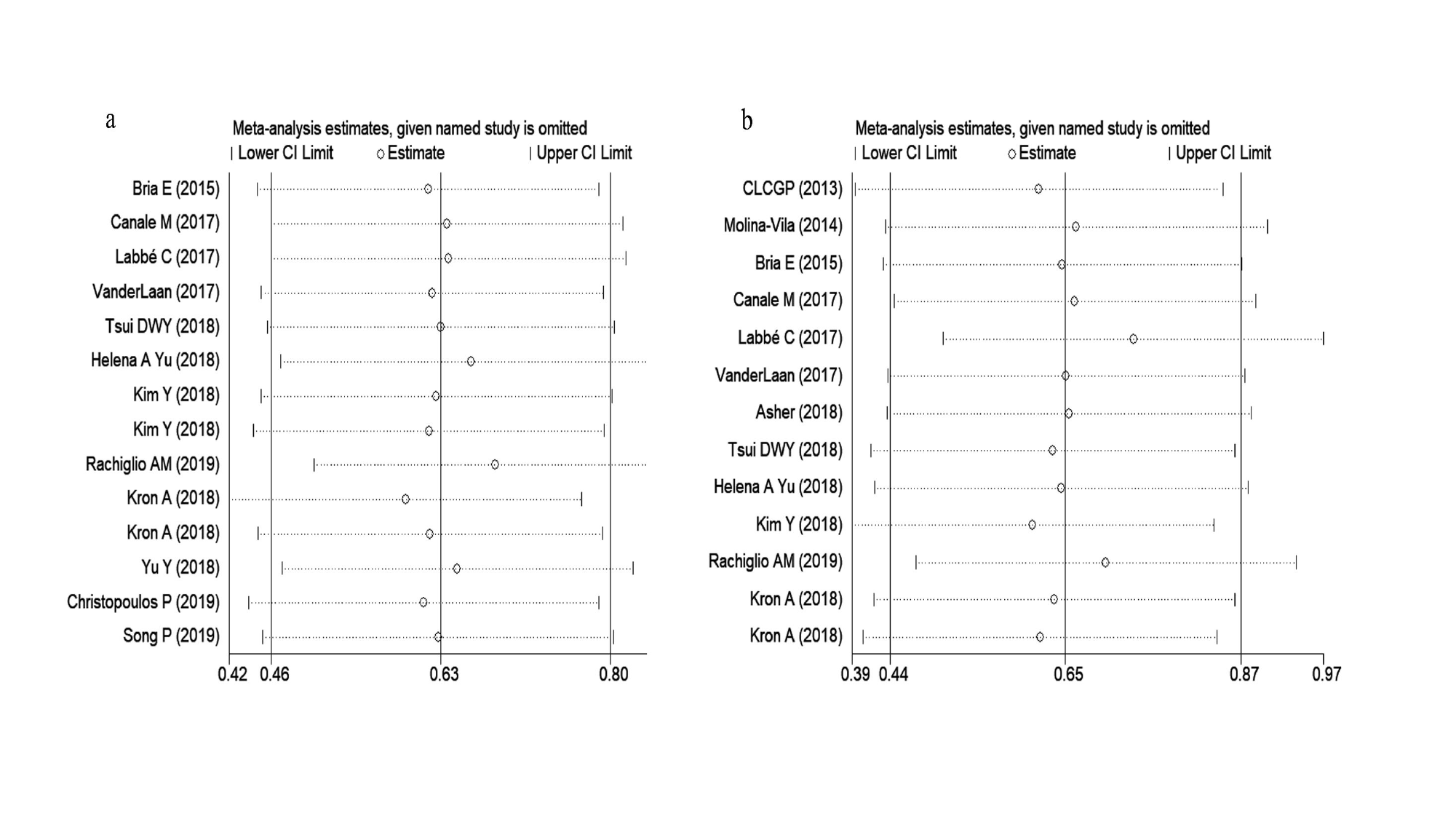

Supplement: Supplementary file 1 — Additional file 1: Figure S1. Sensitivity analyses for (a) overall PFS (b) overall OS. Abbreviations: PFS, progression-free survival; OS, overall survival. [file 12885_2020_6805_MOESM1_ESM.pptx]

## Slide 1
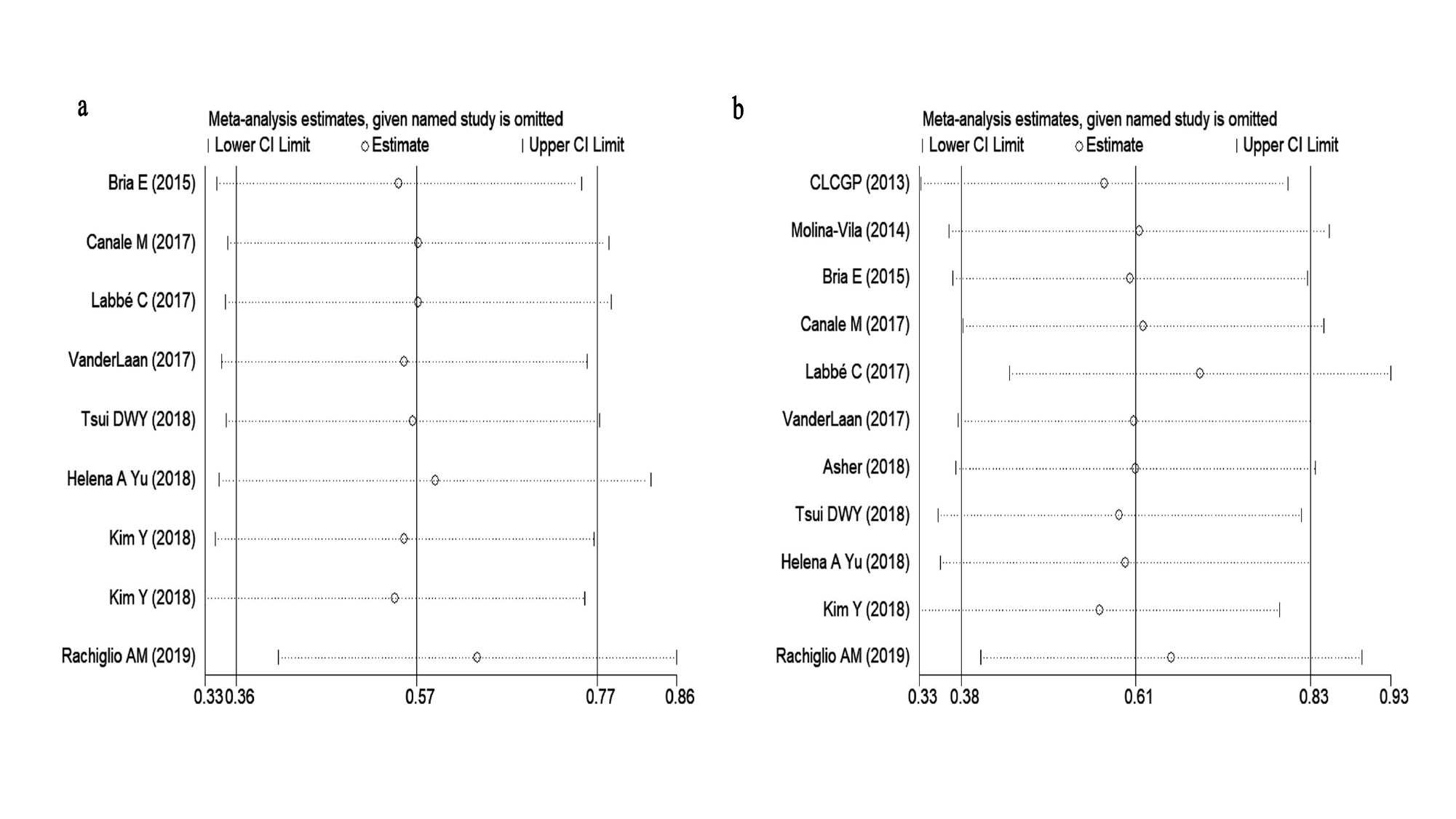

Supplement: Supplementary file 2 — Additional file 2: Figure S2. Sensitivity analyses for (a) PFS (b) OS of patients with EGFR-TKIs treatments. Abbreviations: PFS, progression-free survival; OS, overall survival; EGFR, epidermal growth factor receptor; TKI, tyrosine kinase inhibitor. [file 12885_2020_6805_MOESM2_ESM.pptx]

## Slide 1
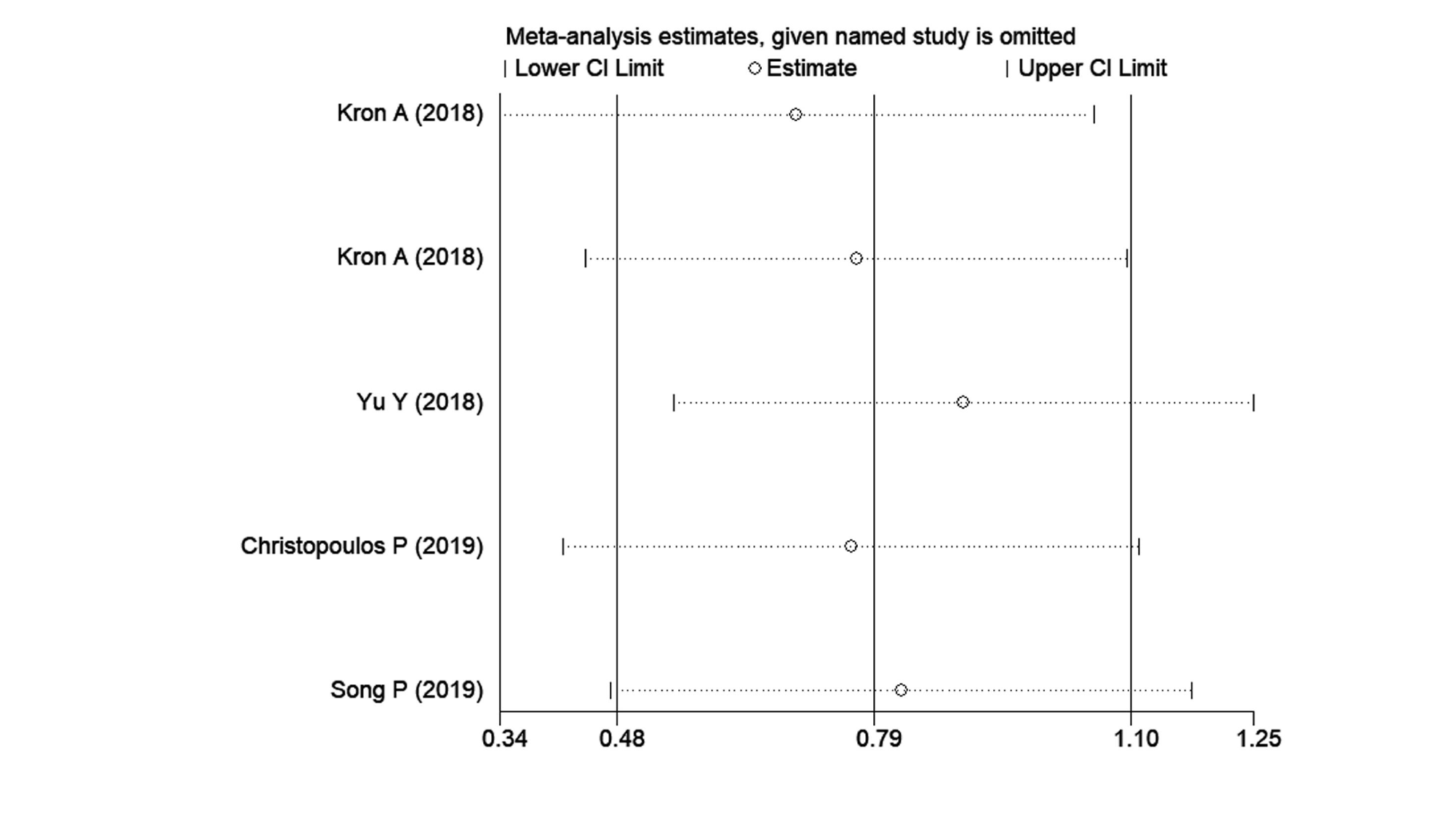

Supplement: Supplementary file 3 — Additional file 3: Figure S3. Sensitivity analysis of PFS for patients with ALK-TKIs therapy. Abbreviations: PFS, progression-free survival; ALK, anaplastic lymphoma kinase; TKI, tyrosine kinase inhibitor (PPTX 610 kb) [file 12885_2020_6805_MOESM3_ESM.pptx]

## Slide 1
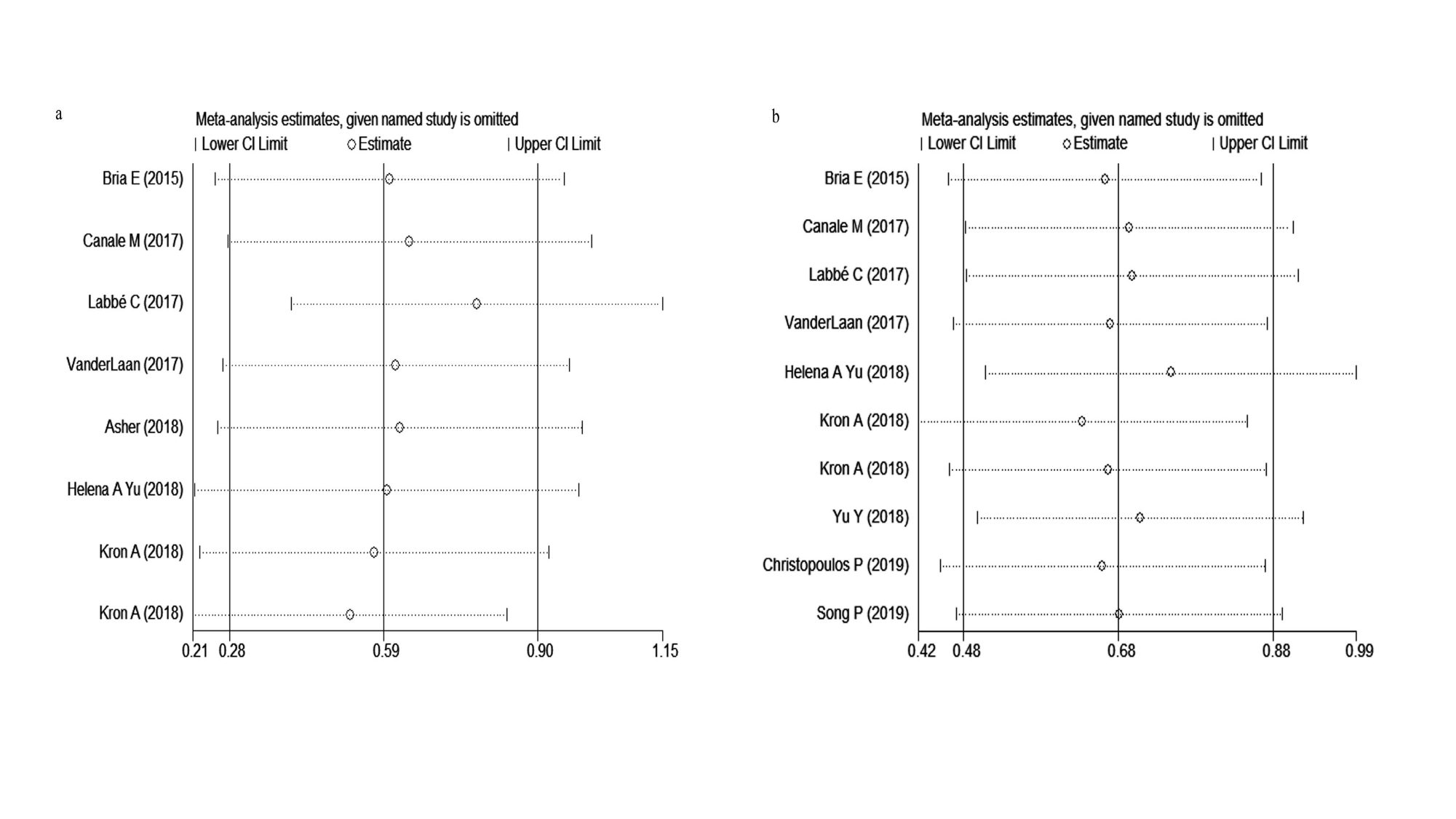

Supplement: Supplementary file 4 — Additional file 4: Figure S4. Sensitivity analyses of (a) PFS and (b) OS in patients with ADC Abbreviations: PFS, progression-free survival; OS, overall survival; ADC, adenocarcinoma. [file 12885_2020_6805_MOESM4_ESM.pptx]

## Slide 1
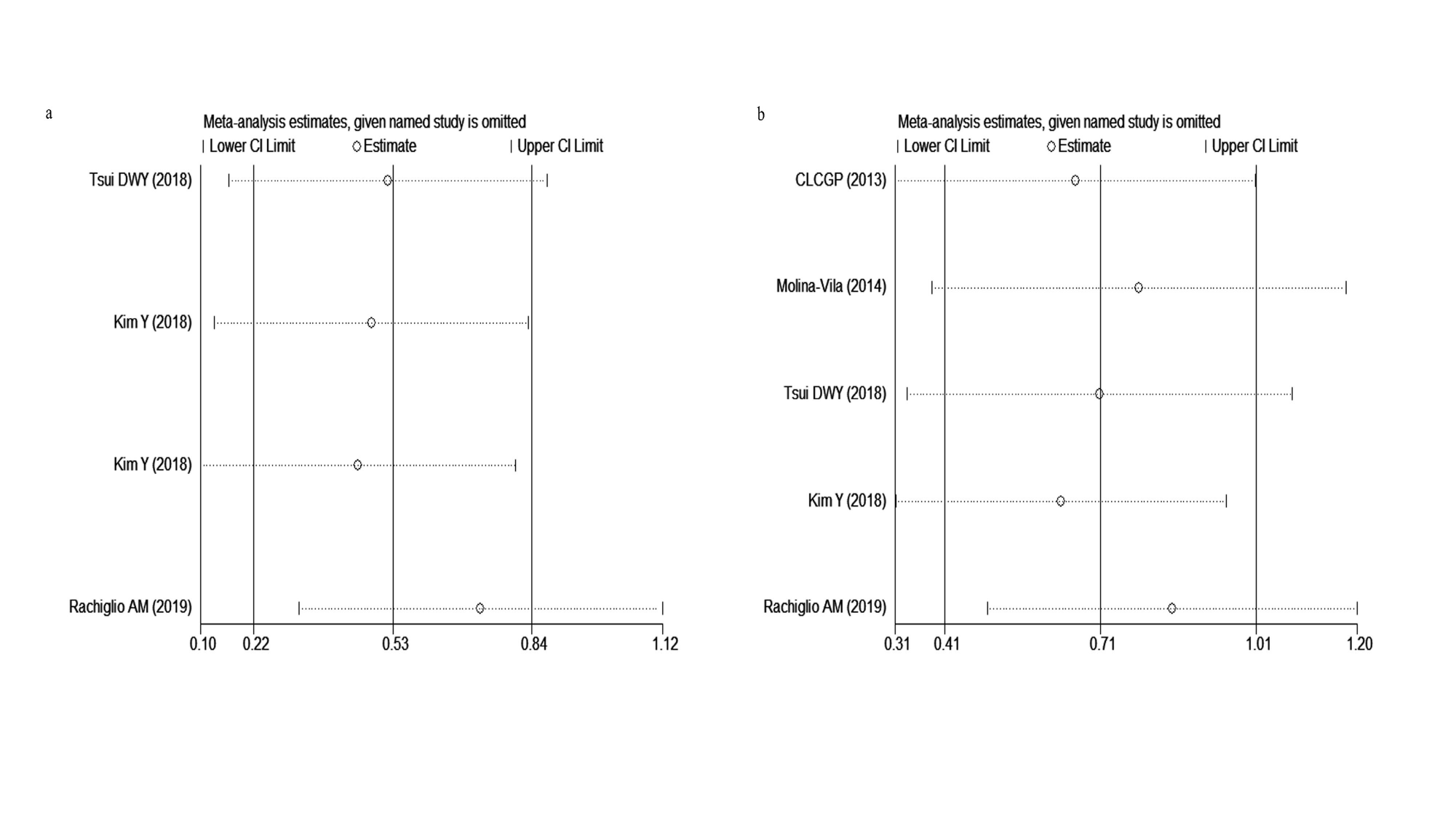

Supplement: Supplementary file 5 — Additional file 5: Figure S5. Sensitivity analyses of (a) PFS and (b) OS in patients with NSCLCs. Abbreviations: PFS, progression-free survival; OS, overall survival; NSCLC, non-small cell lung cancer (PPTX 871 kb) [file 12885_2020_6805_MOESM5_ESM.pptx]

## Slide 1
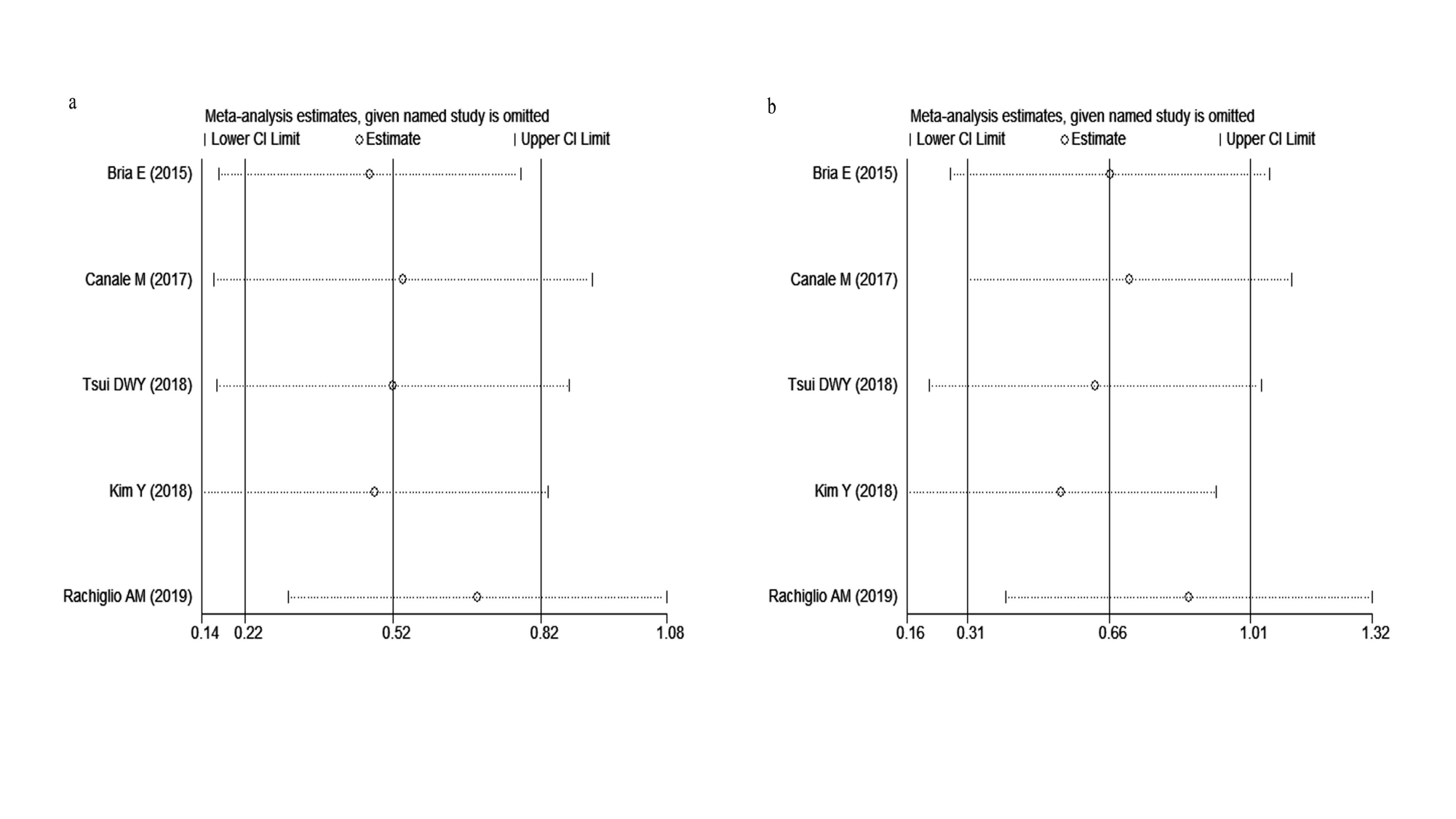

Supplement: Supplementary file 6 — Additional file 6: Figure S6. Sensitivity analyses of (a) PFS and (b) OS in patients with first line EGFR-TKIs or ALK-TKIs treatments. Abbreviations: PFS, progression-free survival; OS, overall survival; NSCLC, non-small cell lung cancer. [file 12885_2020_6805_MOESM6_ESM.pptx]

## Slide 1
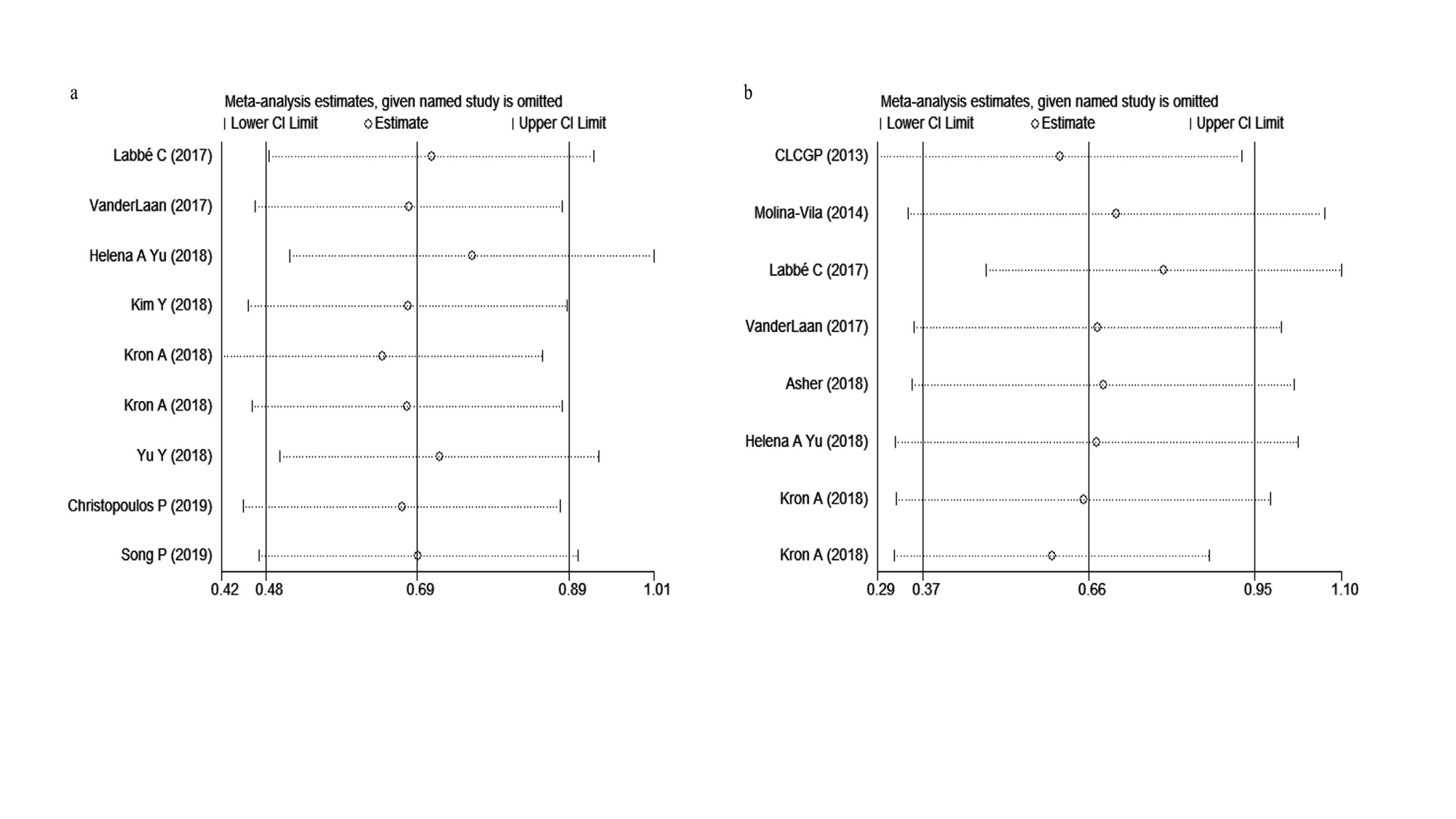

Supplement: Supplementary file 7 — Additional file 7: Figure S7. Sensitivity analyses of (a) PFS and (b) OS in patients EGFR-TKIs or ALK-TKIs treatments in all lines setting. Abbreviations: PFS, progression-free survival; OS, overall survival; EGFR, epidermal growth factor receptor; ALK, anaplastic lymphoma kinase; TKI, tyrosine kinase inhibitor. [file 12885_2020_6805_MOESM7_ESM.pptx]

## Slide 1
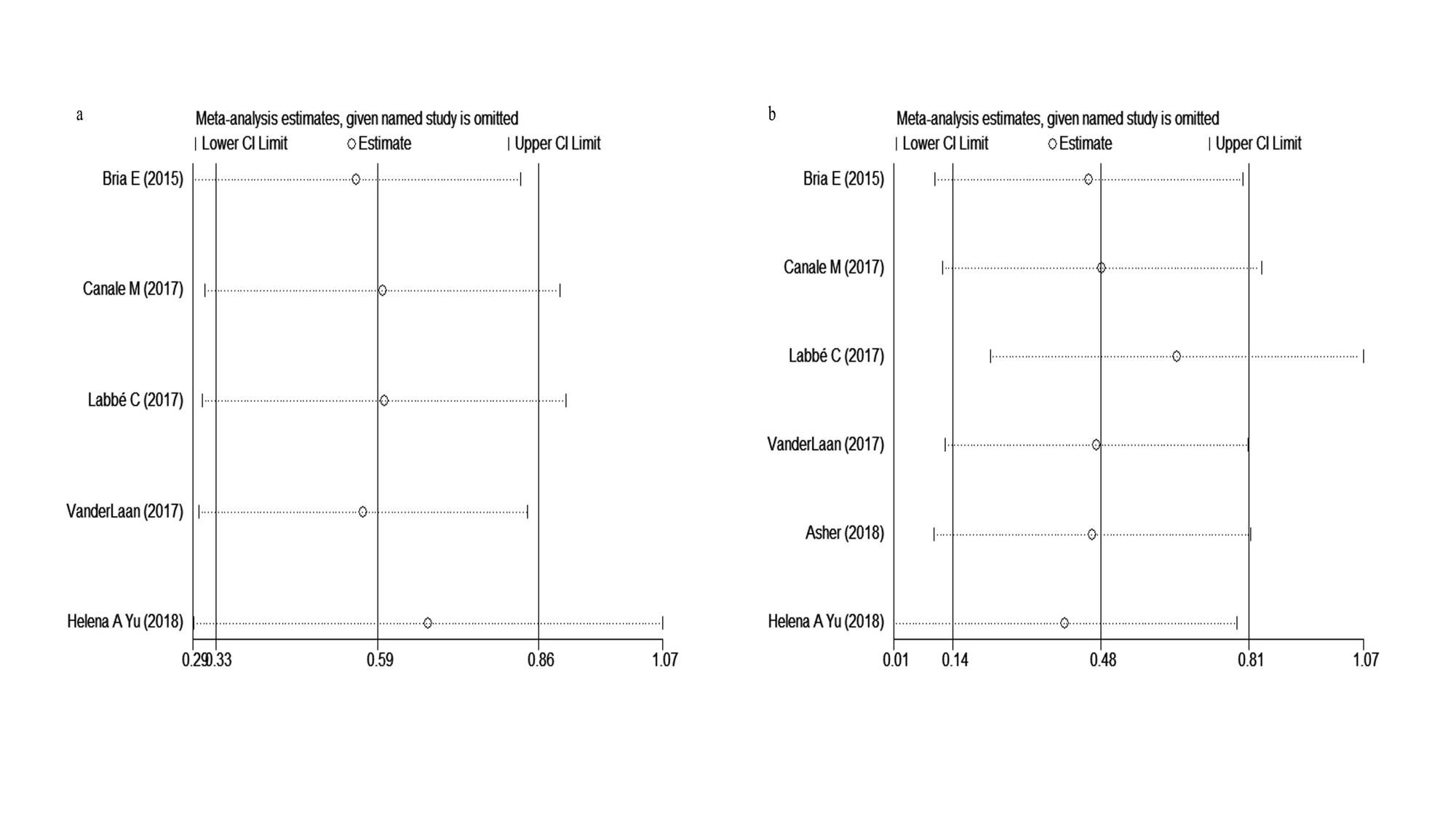

Supplement: Supplementary file 8 — Additional file 8: Figure S8. Sensitivity analyses of (a) PFS and (b) OS in ADC patients with EGFR-TKIs treatments. Abbreviations: PFS, progression-free survival; OS, overall survival; ADC, adenocarcinoma; EGFR, epidermal growth factor receptor; TKI, tyrosine kinase inhibitor. [file 12885_2020_6805_MOESM8_ESM.pptx]

## Slide 1
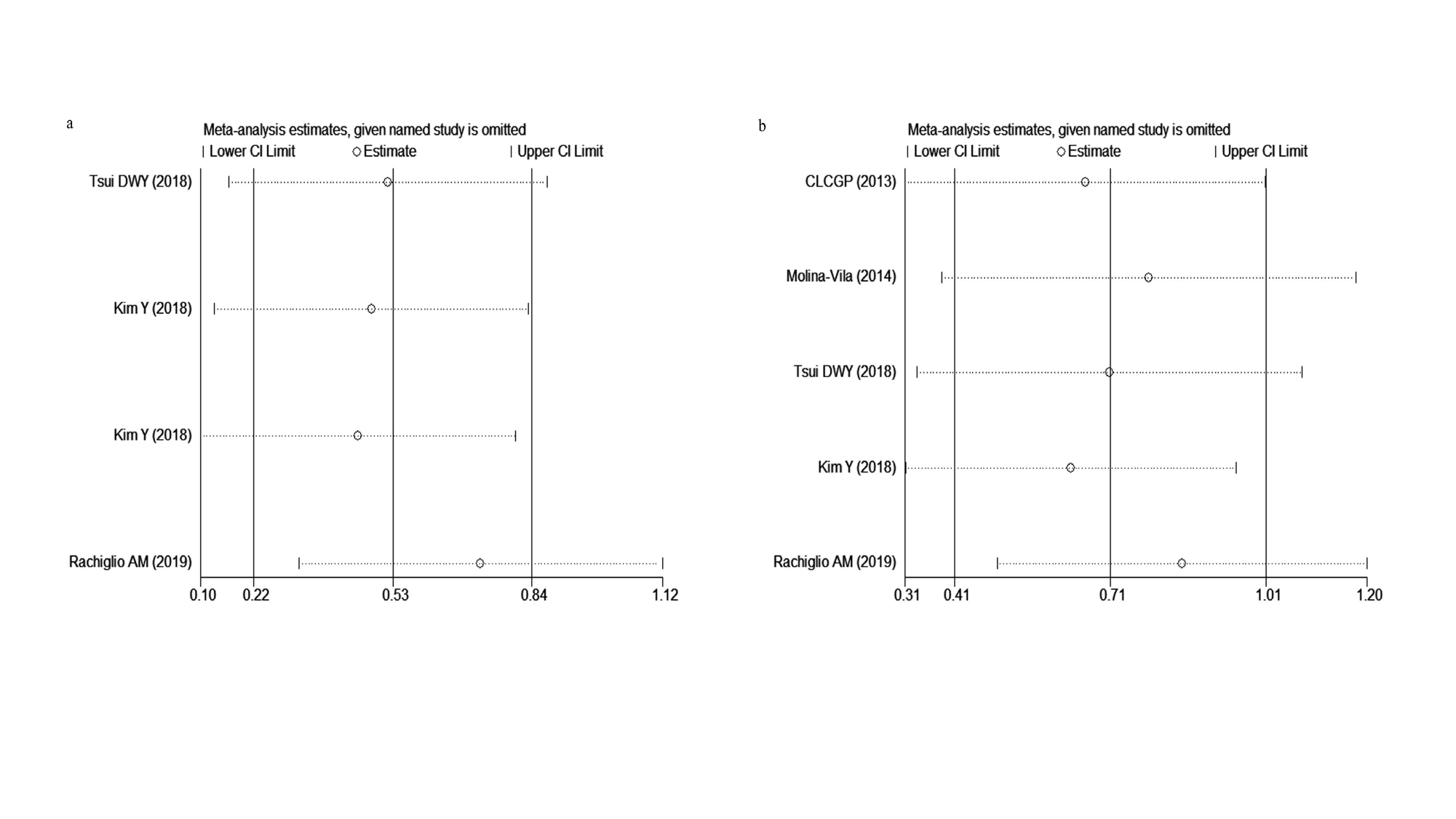

Supplement: Supplementary file 9 — Additional file 9: Figure S9. Sensitivity analyses of (a) PFS and (b) OS in NSCLC patients with EGFR-TKIs treatments. Abbreviations: PFS, progression-free survival; OS, overall survival; NSCLC, non-small cell lung cancer; EGFR, epidermal growth factor receptor; TKI, tyrosine kinase inhibitor. [file 12885_2020_6805_MOESM9_ESM.pptx]

## Slide 1
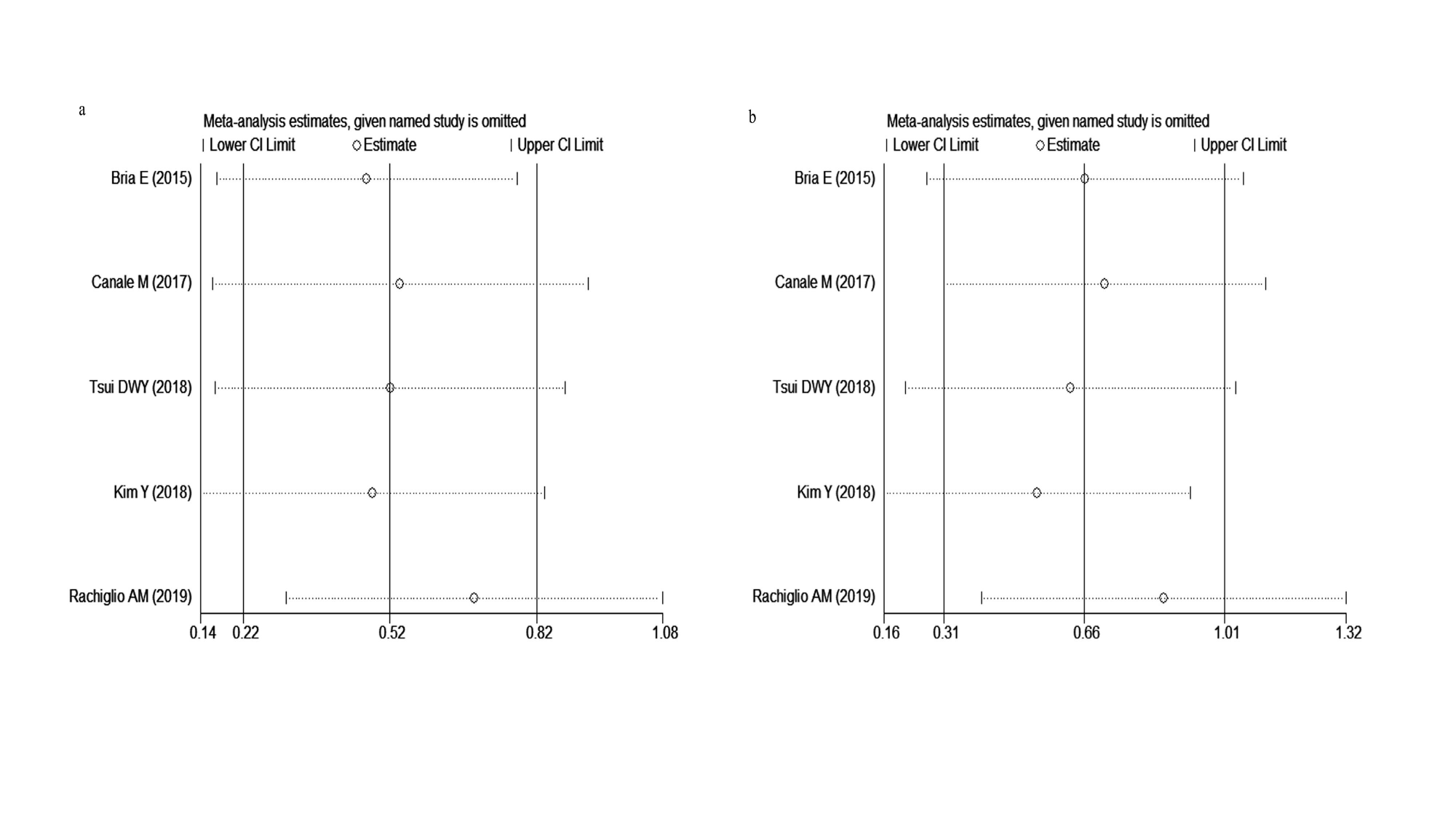

Supplement: Supplementary file 10 — Additional file 10: Figure S10. Sensitivity analyses of (a) PFS and (b) OS in patients with first line EGFR-TKIs treatments. Abbreviations: PFS, progression-free survival; OS, overall survival; EGFR, epidermal growth factor receptor; TKI, tyrosine kinase inhibitor. [file 12885_2020_6805_MOESM10_ESM.pptx]

## Slide 1
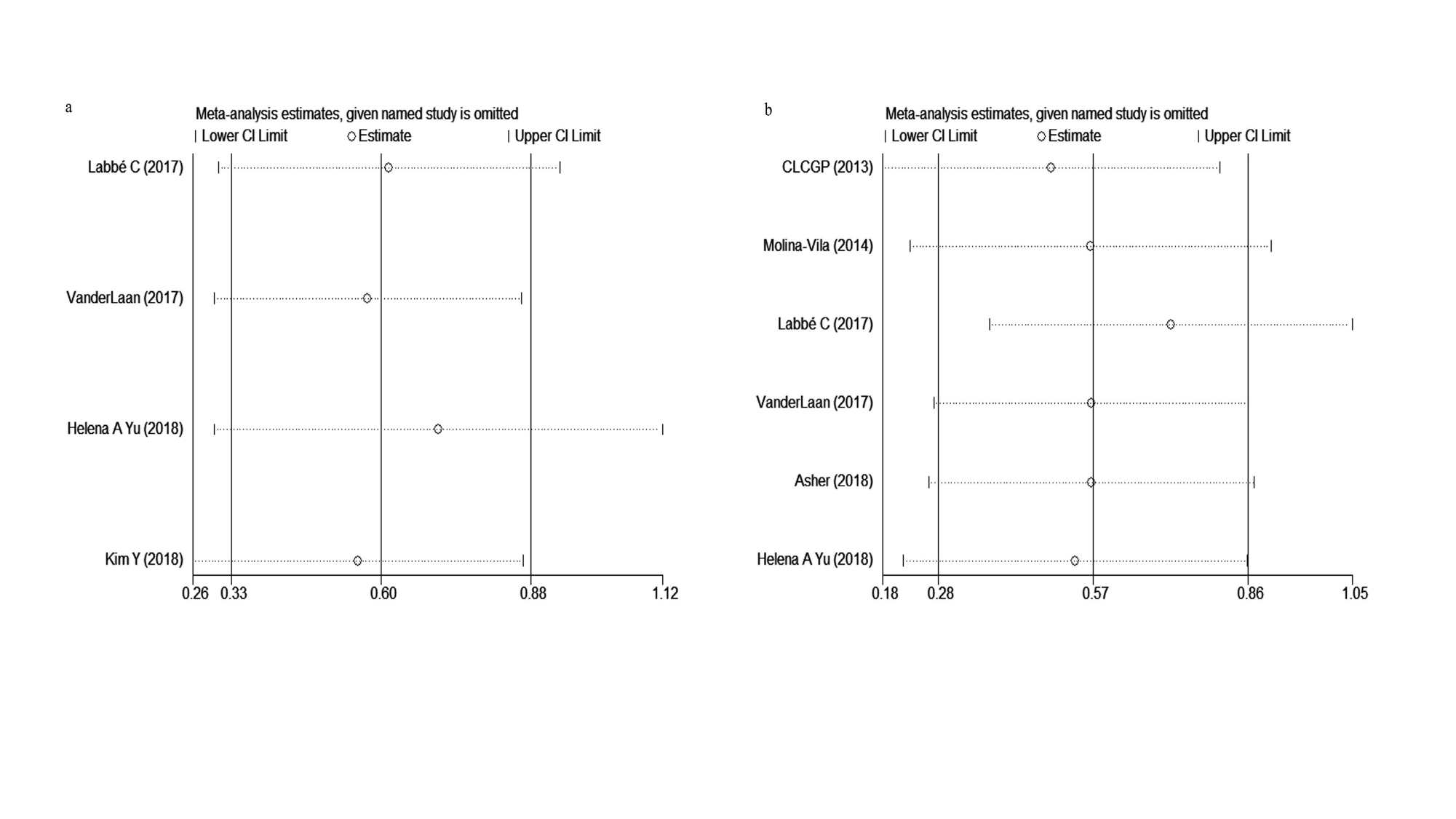

Supplement: Supplementary file 11 — Additional file 11: Figure S11. Sensitivity analyses of (a) PFS and (b) OS in patients with EGFR-TKIs treatments in all-lines setting. Abbreviations: PFS, progression-free survival; OS, overall survival; EGFR, epidermal growth factor receptor; TKI, tyrosine kinase inhibitor. [file 12885_2020_6805_MOESM11_ESM.pptx]
